# Rescuing epileptic and behavioral alterations in a Dravet syndrome mouse model by inhibiting eukaryotic elongation factor 2 kinase (eEF2K)

**DOI:** 10.1186/s13229-021-00484-0

**Published:** 2022-01-03

**Authors:** Stefania Beretta, Laura Gritti, Luisa Ponzoni, Paolo Scalmani, Massimo Mantegazza, Mariaelvina Sala, Chiara Verpelli, Carlo Sala

**Affiliations:** 1 CNR Neuroscience Institute, Milan, and NeuroMi Milan Center for Neuroscience, Via Raoul Follereau 3, 20854 Vedano al Lambro, MB Italy; 2 L’Unità Operativa Complessa di Epilettologia Clinica e Sperimentale, Foundation Istituto di Ricerca e Cura a Carattere Scientifico (IRCCS), Neurological Institute Carlo Besta, 20133 Milan, Italy; 3 CNRS UMR 7275, Institut National de La Santé Et de La Recherche Médicale, LabEx ICST, Institute of Molecular and Cellular Pharmacology (IPMC), Université Côte d’Azur (UCA), 06560 Valbonne-Sophia Antipolis, France

**Keywords:** Inhibitory synapses, Protein translation, EEG, *SCN1A* gene

## Abstract

**Background:**

Dravet Syndrome is a severe childhood pharmaco-resistant epileptic disorder mainly caused by mutations in the *SCN1A* gene, which encodes for the α1 subunit of the type I voltage-gated sodium channel (Na_V_1.1), that causes imbalance between excitation and inhibition in the brain. We recently found that eEF2K knock out mice displayed enhanced GABAergic transmission and tonic inhibition and were less susceptible to epileptic seizures. Thus, we investigated the effect of inhibition of eEF2K on the epileptic and behavioral phenotype of Scn1a ± mice, a murine model of Dravet Syndrome.

**Methods:**

To elucidate the role of eEF2K pathway in the etiopathology of Dravet syndrome we generated a new mouse model deleting the eEF2K gene in Scn1a ± mice. By crossing Scn1a ± mice with eEF2K−/− mice we obtained the three main genotypes needed for our studies, Scn1a+/+ eEF2K+/+ (WT mice), Scn1a ± eEF2K+/+ mice (Scn1a ± mice) and Scn1a ± eEF2K−/− mice, that were fully characterized for EEG and behavioral phenotype. Furthermore, we tested the ability of a pharmacological inhibitor of eEF2K in rescuing EEG alterations of the Scn1a ± mice.

**Results:**

We showed that the activity of eEF2K/eEF2 pathway was enhanced in Scn1a ± mice. Then, we demonstrated that both genetic deletion and pharmacological inhibition of eEF2K were sufficient to ameliorate the epileptic phenotype of Scn1a ± mice. Interestingly we also found that motor coordination defect, memory impairments, and stereotyped behavior of the Scn1a ± mice were reverted by eEF2K deletion. The analysis of spontaneous inhibitory postsynaptic currents (sIPSCs) suggested that the rescue of the pathological phenotype was driven by the potentiation of GABAergic synapses.

**Limitations:**

Even if we found that eEF2K deletion was able to increase inhibitory synapses function, the molecular mechanism underlining the inhibition of eEF2K/eEF2 pathway in rescuing epileptic and behavioral alterations in the Scn1a ± needs further investigations.

**Conclusions:**

Our data indicate that pharmacological inhibition of eEF2K could represent a novel therapeutic intervention for treating epilepsy and related comorbidities in the Dravet syndrome.

**Supplementary Information:**

The online version contains supplementary material available at 10.1186/s13229-021-00484-0.

## Background

Dravet syndrome, also known as Severe Myoclonic Epilepsy in Infancy (SMEI), is a severe childhood disorder that typically presents in the first years of life with pharmacoresistant epilepsy followed by devastating effects on cognitive development. Dravet syndrome typically presents with febrile seizures that later develop to severe partial or generalized tonic–clonic seizures, myoclonic seizures, atypical absences, and focal seizures, as well as episodes of status epilepticus [1–3]. From the second year of life patients develop important comorbidities such as cognitive impairment, including intellectual disability and autistic traits [4–6], behavioral disturbances, including psychomotor delay, ataxia, sleep disorder, impairment in visuospatial and language development [7, 8], and they also have higher incidence of premature death. Approximately 80% of patients with Dravet syndrome carry mutations in the *SCN1A* gene which encodes for the α1 subunit of the type I voltage-gated sodium channel (Nav1.1) [9, 10]. Mutations associated with Dravet syndrome are randomly distributed along the gene and include missense (40%), nonsense/truncated (40%) and the remaining 20% are frameshift mutations, which can be the result of insertion, duplication and deletion of nucleotide [1, 11, 12]. Na_V_1.1 containing channels, despite being broadly expressed in different neurons of the brain including pyramidal neurons, are highly expressed in GABAergic interneurons in hippocampus and cerebral cortex. Loss-of-function of Na_V_1.1 and the consequent reduction in sodium current, greatly impairs the ability of these interneurons to fire action potentials at high frequency and therefore reduces their phasic release of GABA [1, 13–15]. This results in an imbalance between excitation and inhibition that leads to hyperexcitability and seizures [1] and suggests that a promising therapy for Dravet syndrome might be based on normalizing this excitation/inhibition unbalance.

eEF2K, previously known as calcium/calmodulin-dependent protein Kinase III (CaMKIII), is a ubiquitous protein kinase involved in the control of mRNA translation, whose catalytic activity is Ca^2+^-dependent. Upon activation, eEF2K phosphorylates and inhibits eukaryotic elongation factor 2 (eEF2), leading to inhibition of mRNA translation at the level of elongation [16, 17]. We recently demonstrated that eEF2K deletion in mice caused an enhancement in GABAergic transmission that was accompanied by an increased resistance to epilepsy [18]. Accordingly, we showed that eEF2K deletion, in a mouse model of human epilepsy, the Synapsin1 knock out mice, rescued the epileptic phenotype [18].

In this study we investigated the effect of inhibition of eEF2K on the epileptic and behavioral phenotype of Scn1a ± mice, a murine model of DS [13, 19]. Our results demonstrate that genetic deletion of eEF2K in Scn1a ± mice rescued both electroencephalography (EEG) and behavioral alterations. Additionally, we show that pharmacological inhibition of eEF2K ameliorated the altered EEG phenotype, demonstrating that treatments aimed to decrease eEF2K activity might represent a new approach for treating patients that are affected by Dravet syndrome.

## Methods

### Mice

Scn1a ± eEF2K−/− mice were generated by crossing Scn1a ± mice [13] with eEF2K−/− mice [20] and backcrossed for about 20 generations before were used for all the experiments. Mice were housed under constant temperature (22 ± 1 °C) and humidity (50%) conditions with a 12 h light/dark cycle and were provided with food and water ad libitum. All the experiments were performed on female and male mice. All experiments involving animals followed protocols in accordance with the guidelines established by the European Communitis Council and the Italian Ministry of Health (Rome, Italy) for the correct use of laboratory animals in research. Experimental procedures of EEG and behavioral analysis followed the guidelines established by the Italian Council on Animal Care and were approved by the Italian Government decree No. 17/2013 and 980/2017. All efforts were made to minimize the number of subjects used and their suffering.

#### Mice genotyping

All primers were provided from Thermo Fisher and the REDExtract-N-Amp PCR Reaction Mix™ reagent used for the polymerase reaction was provided from Sigma-Aldrich®. In order to discriminate WT and mutated allele of eEF2K gene, two different and separate PCR reactions were used with two different sets of primers. In one PCR mix we used a set of primers SA8/SA5 (5′-GGCCGGCTGCTAGAGAGTGTC-3′ and 5′-CATCAGCTGATTGTAGTG GACATC-3′) that specifically amplify a 1.2 kb band only in WT allele of eEF2K gene. In a separate second PCR mix the set of primers SA8/Neo1 (5′-GGCCGGCTGCTAGAGAGTGTC-3′ and 5′-TGCGAGGCCAGAGGCCACTTGTGTAGC-3′) was used to specifically amplify a band of 1.2 kb only in the mutated allele of eEF2K gene [21]. PCR for SCN1A was performed using the following primer: 5′-CGAATCCAGATGGAAGAGCGGTTCATGGCT-3′, 5′-ACAAGCTGCTATGGACATTGTCAGGTCAGT-3′ and 5′-TGGCGCTCGATGTTCAGCCCAAGC-3′.

### Protein biochemistry

Mice were sacrificed and hippocampus, cerebral cortex, liver, kidney and heart were harvested and lysated in buffer containing 10 mM Hepes pH 7.4, 2 mM EDTA, protease inhibitors (Sigma, P8340) and phosphatase inhibitors (Roche). Samples were centrifuged at 800 × g for 5 min at 4 °C. Resulting supernatant were collected and quantified by BCA protein assay (EuroClone) to assess protein concentration and then solubilized in 4 × loading dye (250 mM Tris–HCl pH 6.8, 40% glycerol, 0.008% bromophenol blue, 8% SDS; all from Sigma-Aldrich). All samples were boiled at 65 °C for 10 min and then equal amounts of each sample were separated using SDS-PAGE and subsequently blotted on nitrocellulose membranes using the Trans- Blot Turbo System (Bio-Rad). Membranes were washed in Tris-buffered saline-Tween (TBS-T) (20 mM Tris pH 7.4, 150 mM NaCl (both Sigma-Aldrich) and 0.1% Tween 20 (Bio-Rad). After 1 h blocking at room temperature with 5% Bovine Serum Albumin (BSA) or 5% milk in TBS-T, membranes were incubated overnight at 4 °C with primary antibody in blocking buffer (TBS-T containing 3% BSA or 3% milk). The membranes were washed three times in TBS-T and then incubated with HRP-conjugated secondary antibodies in TBS-T and 3% BSA or 3% milk for 1 h at room temperature. After three washes (10 min each), chemiluminescence was induced using an ECL Western Blotting Substrate kit and further detected using a ChemiDoc XRS+ machine. All signals were quantified using ImageLab softwer and normalized against the values of the respective signal for actin, βIII-tubulin and α-tubulin.

### Antibodies

The following primary antibody were used (dilution and source): mouse anti-actin (1:5.000 Sigma-Aldrich), mouse anti-βIII-tubulin (1:10.000 Sigma-Aldrich), mouse anti- α-tubulin (1:10.000 Sigma-Aldrich), rabbit anti-eEF2 (1:500 Cell Signaling), rabbit anti-peEF2 (T56) (1:1.000 Cell Signaling), mouse anti-Akt (1:1.000 Cell Signaling), rabbit anti-pAkt (S473) (1:1.000 Cell Signaling), rabbit anti-synapsin1/2 (1:1.000 Synaptic Systems), mouse anti-GABA_A_Rα5 (1:250 NeuroMab). All HRP-conjugated secondary antibodies were purchased from Jackson ImmunoResearch. HRP anti-rabbit (1:3.000) and anti-mouse (1:5.000) were used for western blot.

### Electrophysiology

#### Preparation of brain slices

Hippocampal slices were prepared from P25–P35 mice using standard procedures [22, 23]. Mice were deeply anesthetized with isoflurane and decapitated. The brain was quickly removed, and horizontal hippocampal slices (300 μm) were cut with a Vibratome in chilled (0–4 °C) slicing solution containing (mM) 75 sucrose, 87 NaCl, 25 NaHCO_3_, 25 D-glucose, 2.5 KCl, 1.25 NaH_2_PO_4_, 0.5 CaCl_2_, 7.0 MgCl_2_. The slices were transferred to a storage chamber and incubated at room temperature for at least 45 min before recording, then transferred to a recording chamber and perfused with ACSF containing (mM) 129 NaCl, 3 KCl, 1.8 MgSO_4_, 1.6 CaCl_2_, 1.25 NaH_2_PO_4_, 21 NaHCO_3_, and 10 D-glucose at 28/30 °C. All solutions were saturated with 95% O_2_ and 5% CO_2_.

#### Brain slice recording

Whole-cell voltage-clamp recordings were performed on CA1 hippocampal pyramidal neurons visualized with near infrared differential interference contrast (DIC) optics. For the recordings of inhibitory postsynaptic currents (IPSCs), the intracellular solution contained (mM) 135 CsCl, 10 N-2-hydroxyethylpiperazine-Nʹ-2-ethanesulfonic acid (HEPES), 0.2 ethylene glycol- bis(β-aminoethylether)-N,N,Nʹ,Nʹ-tetraacetic acid (EGTA), 2 Mg-ATP, 4 ATP, 10 phosphocreatine, pH 7.2 with CsOH. Kynurenic acid (KA, 3 mM) was included in the extracellular recording solution to block the excitatory synaptic transmission in IPSC recordings. The holding potential was -70 mV. Patch pipettes were pulled from borosilicate glass and, when filled with the intracellular solution, their resistance ranged from 3 to 5 MΩ. Recordings were obtained by means of a Multiclamp 700B amplifier, Digidata 1440 digitized and pCLAMP 8.0 software (Molecular Devices). Access resistance was continuously monitored for each cell. Only the cells with access resistance less than 20 MΩ were recorded, and recordings were terminated/discarded when a significant (> 10%) increase occurred. Data were analyzed using Clampfit 10.5 (Molecular Devices) and Mini Analysis (Synaptosot).

### Electroencephalography analysis

For electroencephalography (EEG) a total of 26 animals between P60-P90 were used (4 WT, 17 Scn1a ± , 5 Scna1 ± eEF2K−/−). Animals were divided in two different groups. One group of 4 WT, 7 Scn1a ± and 5 Scna1 ± eEF2K−/− mice was used for electroencephalography analysis under baseline and under thermal stress condition. A second group of animals (5 Scn1a ± for placebo and 5 Scn1a ± for A484954) was used for electroencephalography analysis under pharmacological inhibition of eEF2K by A484954. Mice were anesthetized with isoflurane (2% (v /v) in 1 L/min O2). Four screw electrodes (Bilaney Consultants GMBH, Dusseldorf, Germany) were inserted bilaterally through the skull over the cortex (anteroposterior, + 2.0–3.0 mm; left–right 2.0 mm from bregma), as previously described [24] and in agreement to brain atlas coordinates [25]. Mice were allowed to recover for approximately a week from surgery under antimicrobial cover (Cetriaxone, Sigma-Aldrich; 50 mg/kg i.p.) before performing the experiments. EEG activity was recorded in awake freely moving mice placed in a Faraday chamber using a Power-Lab digital acquisition system (AD Instruments, Bella Vista, Australia; sampling rate 100 Hz, resolution 0.2 Hz).

#### Electroencephalography analysis under baseline conditions

Seven days after surgery basal cerebral activity was recorded continuously for 24 h. All EEG tracings were analyzed and scored for the presence of spikes. EEG spikes were recognized as having a duration < 200 ms with a baseline amplitude set to 4.5 times the standard deviation of the EEG signal (determined during inter-spike activity periods) whereas epileptic discharge was defined as 3 or more spikes lasting < 5 s. Segments with electrical noise or movements artifacts were excluded from statistical analysis [24].

#### Electroencephalography analysis under thermal stress condition

The day after EEG basal activity, mice body temperature was monitored and managed by a rectal temperature probe connected to a controller. Average mouse body temperature is 36.9 °C and it was controlled by a heat lamp above the chamber. Mice were accustomed to the Plexiglas cage for at least 10 min at 37.5 °C. Then, the body temperature was increased by 0.5 °C every 2 min until a tonic–clonic seizure occurred or 42.5 °C was reached [26, 27]. Immediately after the heat stimulus was removed. Seven days after heat EEG basal activity was measured for 24 h and the number of spikes was evaluated.

#### Electroencephalography analysis under pharmacological inhibition of eEF2K by A484954 using Matriz-Driven Delivery (MDD) Pellet System

Two days after monitoring basal cerebral activity for 24 h pellets, releasing A484954 or placebo, were implanted in the lateral side of Scn1a ± mice between ear and shoulder. Pellets methodology is provided by The Innovative Research of America (IRA). This company provides finished products in a ready-to-implant form, utilizing their Matriz-Driven Delivery (MDD) Pellet System, a biodegradable matrix that effectively and continuously releases the active product in the animal. The MDD Pellet System has a double process of erosion of the pellet and diffusion of the active product. The pellet is made of a matrix, which is made with cholesterol, cellulose, lactose, phosphates and stearates, fused with an active product. Animals were treated for ten days with 10 mg of A484954/pellets that release 1 mg of drug every day. Placebo pellets were used in experiment as the proper control. During the 10 days of treatment EEG activity was recorded for 24 h after 3, 6 and 9 days of pellets implantation and analyzed as previously described. Animals were sacrificed the last day of the pharmacological treatment.

### Behavioral procedure

Mice of P60-P90 were used for behavioral experiments. Animals were housed in groups of four or five individuals of mixed genotypes. All of the tests were conducted during the light portion of the cycle. All the behavioral experiments followed the ARRIVE guidelines. A total of 190 mice were used for behavioral analyses (60 WT, 70 Scn1a ± , 60 Scna1 ± eEF2K−/−). Animals were divided in two different sets. To minimize the number of animals each mouse was submitted to a maximum of 3 tests with an interval of 1 week between every test. In the first set, animals were divided in 6 different groups and 10 mice per genotype for each group were used for a total number of 60 WT, 60 Scn1a ± and 60 Scn1a ± eEF2K−/−. Animals were divided as follow: Group 1 for elevated plus maze, novel object recognition and tail suspension; Group 2 for self-grooming, wire hanging and spatial object recognition; Group 3 for balance beam, marble burying, and pole test; Group 4 for locomotor activity, sociability/social novelty and rotarod; Group 5 for Morris water maze, acquisition and reversal; Group 6 for T-maze, acquisition and reversal. A second set of animals was used for behavioral analysis under pharmacological inhibition of eEF2K by A484954 (5 Scn1a ± for placebo and 5 Scn1a ± for A484954). In addition, another set of 70 animals (34 WT and 36 eEF2K−/−) was used to investigate sensory abilities, motor coordination, sociability, anxiety- and depression-like behavior and episodic and spatial memory in WT and eEF2K−/− mice. Animals were divided in 3 groups: Group 1 (range of 8–12 animals for genotype) for olfactory and visual cliff test, balance beam and novel object recognition; Group 2 (range of 5–10 animals for genotype) for self-grooming, pole test and spatial object recognition; Group 3C (range of 5–12 animals for genotype) for rotarod test, sociability test and tail Suspension). All the behavioral scoring was performed on a blind basis by a trained experimenter.

#### Locomotor activity

The spontaneous motor activity was evaluated in an automated activity cage (43 × 43 × 32 cm) (Ugo Basile, Varese, Italy), located in a sound-attenuating room as previously described [28]. Horizontal and vertical activity were detected by infrared photobeam break sensors put from 2.5 cm and 4 cm from the floor of the cage, respectively. Cumulative horizontal and vertical movements were counted for 3 h.

#### Wire hanging

The limb force was tested by positioning a mouse on the top of a wire cage lid (19 × 29 cm) that was turned upside down at approximately 25 cm above the surface of the bedding material. The grip of the mouse was ensured by gently waving three times before rotating the lid as described in [29]. The latency to fall onto the bedding was registered (cut-off = 300 s).

#### Balance beam walking test

The balance beam walking test is a test for assess motor coordination and balance as previously described [30]. The beam apparatus consisted of 1 m beams with a flat surface of 12 mm width resting 50 cm above the table top on two poles. At the end of the beam a black box was placed as the finish point. Nesting material from home cages was placed in the black box to attract the mouse to the finish point. A lamp (with 60-W light bulb) was used to shine light above the start point and served as an aversive stimulus. A video camera was set on a tripod to record the performance. On training days, each mouse crossed the 12 mm beam 3 times. The time required to cross to the escape box at the other end (80 cm away) was measured with a stopwatch. The stopwatch started when the nose of the mouse began to cross the beam and stopped when the animal reaches the escape box. Once the mice are in the safe box, they are allowed some time (~ 15 s) to rest there. Before the next trial the mice rest for 10 min in their home cages between training sessions on the two beams. On the test day, times to cross each beam were recorded. Two successful trials in which the mouse did not stall on the beam are averaged. The beams and box were cleaned of mouse droppings and wiped with towels soaked with 70% ethanol and then water before the next beam was placed on the apparatus.

#### Pole test

In the pole test, which evaluated motor coordination, mice were trained for 2 days (in the morning and in the afternoon) to descend a vertical pole (90 cm length, 1 cm diameter). Every training consisted of three trials. Mice were accustomed to the room 20 min before trials and test day. The test was performed the third day and the time necessary to the mice to descend the pole in five trials was recorded. A cut-off of 60 s was given [31], with minor modifications. Data were shown as mean of 5 trials evaluated during the test day.

#### Rotarod

The rotarod was used to measure fore and hindlimb motor coordination and balance. During the training period, each mouse was placed on the rotarod apparatus (Ugo Basile, Biological Research Apparatus, Varese, Italy) at a constant speed 12 rpm for a maximum of 120 s, and the latency to fall off the rotarod within this time period was recorded. Mice received four trials per day for 4 straight days.

#### Repetitive self-grooming

Mice were assessed for spontaneous self-grooming as a measure of repetitive behavior as previously described in [32]. Each mouse was placed into a standard plastic cylinder (46 × 23.5 × 20 cm). Cylinder was empty to eliminate digging in the bedding, which is a potentially competing behavior. The room was illuminated at about 40 lx. A front-mounted CC TV camera (Security Cameras Direct) was placed at about 1 m from the cages to record the sessions. Sessions were video-taped for 20 min. The first 10 min of habituation was not scored. In the next 10 min mice were scored with a stopwatch for cumulative time spent grooming all the body regions. The cylinder was cleaned with 70% ethanol between each animal.

#### Elevated plus maze

The Elevated Plus Maze paradigm was used to study anxiety related behavior. The apparatus had a central platform (10 × 10 cm) from which originated two opposite open arms (30 × 10 cm) and two enclosed arms (30 × 10 × 14 cm) according to [33]. The apparatus was made of white wood, placed to a height of 60 cm above floor level in the center of a small quiet room under dim light (about 30 lx). The test was conducted in the morning, during the early light phase of the light cycle. After 20 min of familiarization to the novel environment, mice were placed individually onto the center of the apparatus, facing an open arm. Experimenter recorded for 5 min the number of open- and closed-arm entries and the time spent in open- and closed-arms.

#### Marble burying

Mouse marble burying can be associated to both anxiety-like traits, increased by novelty, and obsessive/compulsive-like behavior as previously described in literature [34]. The marble burying assay is applied to evaluate how many novels glass marbles a rodent would bury. This behavior was assessed using a clean cage (50 × 25 × 30 cm) filled with 3 cm bedding, lightly tamped down to make a flat, even surface. Each mouse was placed individually into the corner of the empty cage where previously food marbles were placed covered by the bedding. The latency to the first marble buried and the number of marbles buried (to 2/3 their depth) with bedding were recorded over a maximum period of 900 s.

#### Tail suspension

Tail suspension test assayed depressive-like behaviors of mice as described in [35]. Tail suspension test was performed on an apparatus with a support at 35 cm from the basis where was fixed a hook where mouse tail was fasten at about 1 cm from the origin. The test was preceded by a familiarization phase where mice were let in the room test at least 1 h before. The test day lasted 6 min during which the experimenter measured time of mouse immobility.

#### Novel Object Recognition test

The novel-object recognition test was performed over 3 straight days in an open plastic arena (60 × 50 × 30 cm) as previously described [36]. The test had 3 phases. In the habituation one, the first day, mice were habituated to the empty arena for 10 min, the familiarization and novel-object recognition the day after. In the familiarization phase, two identical objects were placed in the middle of the arena equidistant from the walls and from each other. Mice were placed between the two objects until it had completed 30 s of cumulative object exploration (20 min cut-off). Object recognition was measured when each animal was within approximately 1 cm of an object with its nose toward the object. Climbing the object or pointing the nose toward ceiling near the object were not considered exploring behaviors. After familiarization, mice were returned to the home cage until they were tested for novel recognition after 5 min, 120 min or 24 h. In the novel recognition phase, a novel object (never seen before) took the place of one of the two familiars. Scoring of object recognition was performed as during the familiarization phase. For each mouse, the role (familiar or new object) as well as the relative position of the two objects were randomly permuted. The objects used for the test was white plastic cylinders and colored plastic Lego stacks of different shapes. The arena was cleaned with 70% ethanol after each trial. Performance was analyzed by calculating a discrimination index (N-F/N + F), where N = the time spent exploring the novel object, and F = the time spent exploring the familiar object.

### Spatial object recognition test

Spatial object recognition test was performed in an arena according to [37], with minor modifications that consisted in an opaque white plexiglass cage (58 × 50 × 43 cm) that was dimly lit from above (27 lx) and two visual cues were placed above two adjacent walls. In the center of the northern wall there was a black and white stripped pattern (21 × 19.5 cm) and in the center of the western wall there was a black and gray checkered pattern (26.5 × 20 cm). The objects were placed across the visual cues. Mice were habituated to the arena for 10 min the day before the test. The test day, first mice were allowed to familiarise with two different objects. The experimenter measured the time spent in sniffing both objects until the mouse completed 30 s in exploring objects (cut-off 20 min). Exploring behavior was defined as mouse having its nose directed toward the object and within approximately 1 cm of the object [38]; climbing or sitting were not considered exploration behaviors. After 5 min, 120 min and 24 h from familiarization phase mice were allowed to re-explore the cage where one object was moved in a new position. Scoring of object recognition was performed as during the familiarization phase. Between two sessions, mice returned to their home-cage. Cage and object were carefully cleaned with 70% ethanol before and after all behavioral procedures. Performance was analyzed by calculating a discrimination index (N-F/N + F), where N = the time spent exploring the moved object during the test and F = the time spent exploring the unmoved object during the test.

#### T-maze

Mice were deprived of food until they reached 90% of their free-feeding body weight. Mice were habituated to a black wooden T-maze (with a 41 cm stem section and a 91 cm arms section, and each section was 11 cm wide and had walls that were 19 cm high) and trained to obtain food within the maze for 5 days as previously described [39]. During the acquisition phase, one arm was designated to be reinforced with Coco Pops (Kellogg’s) in each of ten daily trials. Each mouse was placed at the start of the maze and allowed to freely move and choose which arm to enter. The number of days required to reach the goal criterion (80% correct for 3 days) was recorded. Each mouse that met the goal for acquisition was then tested using a reversal procedure in which the reinforce was switched to the opposite arm. A cut off of 20 days in both acquisition and reversal phase was established.

#### Morris water maze

The Morris water maze test was used to analyze changes in the learning and memory abilities of the mice according to the methods described in Morris [40] (adapted for mice). The Morris water maze consisted of a circular water maze (120 cm in diameter × 50 cm in height) filled with water. The pool was divided into 4 quadrants. A circular hidden platform with a diameter of 10 cm was placed inside the maze, and its surface was maintained at 0.5 cm below the surface of the water.

Floating plastic particles were placed on the surface of the water to hide the platform from sight according to the methods of [41]. During the habituation trials, mice were placed in a random area inside the maze and allowed to swim for 60 s. During the acquisition trials, mice were given 4 daily trials (with 60 min inter-trial intervals) for 4 straight days in which each mouse empty wire cage was released into the pool at different starting points and trained to locate a constant platform position. At 24 h after the last trial, a probe test was performed in which the platform was removed. Two days later, a reversal task was performed to assess cognitive flexibility. The platform was placed in the opposite quadrant of the maze, and 4 daily trials were performed for 4 straight days. On the fifth day, a probe trial was performed that was similar to that in the acquisition phase. The time spent in the target area and the latency to reaching the target zone were evaluated.

#### Sociability and preference for social novelty test

The sociability tests were performed in a rectangular apparatus in transparent polycarbonate with three-chamber (width = 42.5 cm, height = 22.2 cm, central chamber length = 17.8 cm, and side chamber lengths = 19.1 cm) as previously described in [39]. In the 10 min habituation phase, each mouse was placed in the middle compartment and free to explore all chambers. Then, one side of the compartment was occupied by an unfamiliar mouse and the other contained an empty wire cage. Immediately after sociability test, without cleaning the apparatus, the social novelty test was performed by putting an unfamiliar mouse in the empty wire cage. Every test lasted 10 min in which were measured the time spent exploring each chamber. The data were expressed in sociability index (SI) and social novelty preference index (SNI) as follows: SI = (time exploring novel mouse 1 – time exploring empty cage)/(time exploring novel mouse 1 + time exploring empty cage) and SNI = (time exploring novel mouse 2 – time exploring familiar mouse)/(time exploring novel mouse 2 + time exploring familiar mouse).

### Statistical analyses

Based on the number of comparisons and the pattern of data distribution, appropriate statistical tests were used to analyze the data. Data are expressed as the mean ± SEM or percentage, analyzed for statistical significance, and displayed by Prism 8 software (GraphPad Software). Shapiro–Wilk test was applied to test the normal distribution of experimental data. Normal distributions were compared with one simple t-test, student t-test or ANOVA with appropriate post hoc test. Non-normal distributions were compared with the non-parametric Wilcoxon test, Mann–Whitney test or Kruskal–Wallis test with appropriate post hoc test, as indicated. The accepted level of significance was *p* < 0.05. Statistical analyses for every experiment are described accordingly in the figure legends.

## Results

### Higher level of phosphorylate eEF2 (P-eEF2) in cortex and hippocampus of Scn1a ± mice

We previously demonstrated that in a genetic murine models of epilepsy, the Synapsin1−/− mice, eEF2 phosphorylation in brain is strongly increased suggesting that in these mice the pathway that control eEF2 phosphorylation is altered and this might contribute to the excitation/inhibition unbalance and epileptic phenotype [18]. Thus, we wondered if the level of phosphorylated eEF2 (P-eEF2) is also altered in Scn1a ± mice.

**Fig. 1:**
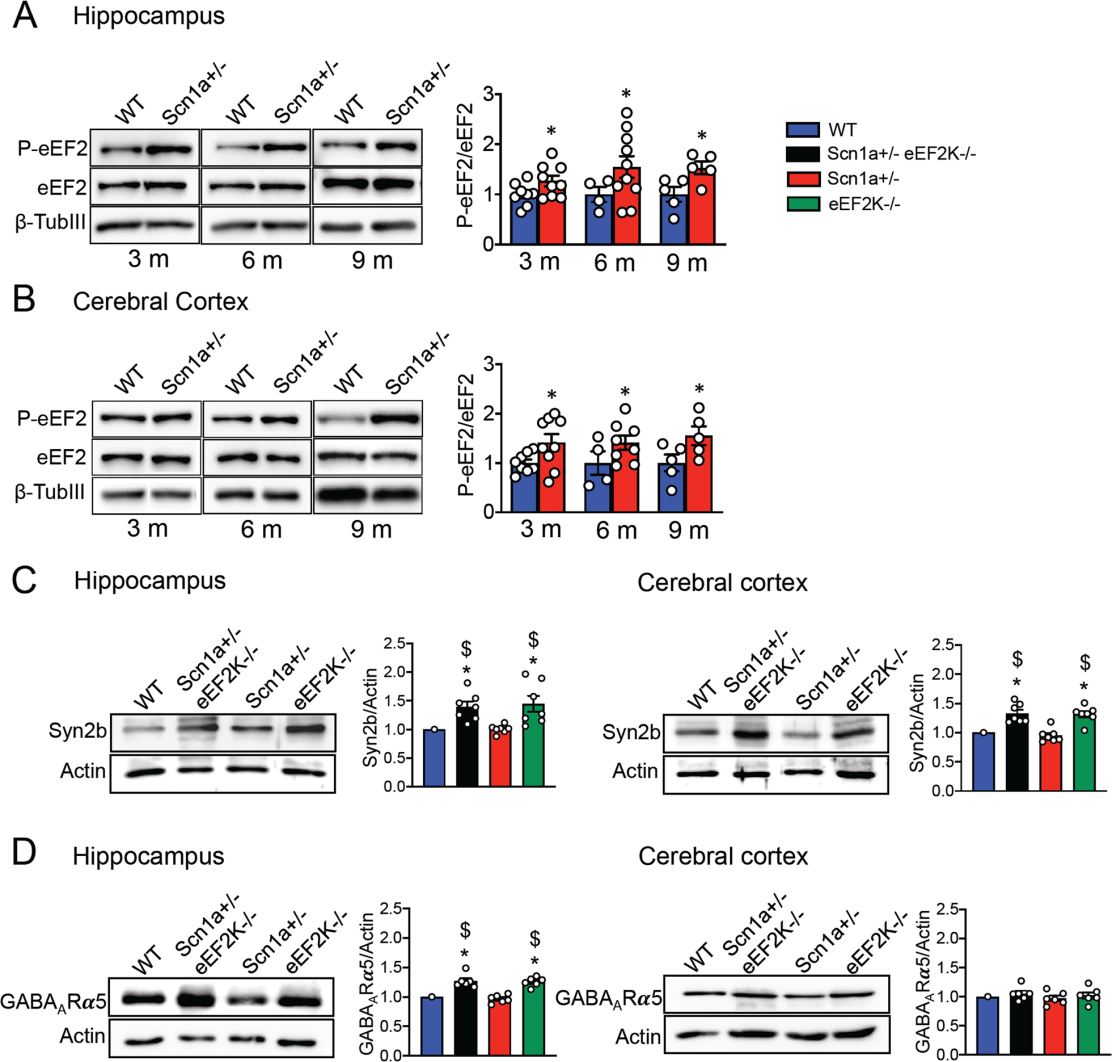
Scn1a ± mice exhibit higher levels of eEF2 phosphorylation in total homogenate of hippocampus and cerebral cortex compared with WT mice and eEF2K deletion modulates the expression of a set of proteins that regulate GABAergic synaptic transmission.** A,B** Representative western blots and relative quantification for phosphorylated eEF2 in samples from hippocampus **(A)** and cerebral cortex **(B)** in 3, 6 and 9 months old Scn1a ± and WT mice. 3 months: hippocampus and cerebral cortex WT n = 8, Scn1a ± n = 9; 6 months: hippocampus WT n = 4, Scn1a ± n = 10, cerebral cortex WT n = 4, Scn1a ± n = 8; 9 months hippocampus and cerebral cortex WT n = 5, Scn1a ± n = 5. All Data are presented as mean ± SEM. Statistical analysis *p < 0.05 versus corresponding WT; One-sample *t*-test. **C,D** Representative western blots and relative quantification show expression levels of Synapsin 2b **(C)** and GABA_A_Rα5 **(D)** in total homogenate of hippocampus (left) and cerebral cortex (right) from 3 months old WT, Scn1a ± eEF2K−/−, Scn1a ± and eEF2K−/− mice. All data are presented as mean ± SEM. N = 7 per group for Synapsin 2b. N = 6 per groups for GABA_A_Rα5. Statistical analysis *p < 0.05 versus corresponding WT, $*p <* 0.05 versus corresponding Scn1a ± ; One-simple *t*-test

We analyzed the amount of P-eEF2 phosphorylation in total homogenate of two main brain areas: hippocampus and cerebral cortex. We collected brain samples at different ages (3, 6 and 9 months) in order to detect eventual changes of P-eEF2 in ageing. Western blots analysis showed that the level of P-eEF2 is significantly increased in Scn1a ± mice at all age analyzed (with no significant changes among ages) both in hippocampus and cerebral cortex (Fig. 1a, b) while the level of P-eEF2 was not altered in other tissue such as liver, kidney and heart (Additional file 1: Figure S 1A–C). These data suggest that the specific alteration of the pathway that control eEF2 in brain areas might further contribute to the imbalance of excitation over inhibition occurred in in these mice as we observed in the Synapsin1−/− mice [18].

### Generation of Scn1a ± eEF2K−/− mice

To elucidate the role of eEF2K pathway in the etiopathology of Dravet syndrome we deleted the eEF2K gene in Scn1a ± mice that was previously generated and described in Yu et al. [13] and mimic the Dravet syndrome described in human patients. We crossed Scn1a ± mice with eEF2K−/− mice (generated by the laboratory of Prof. Alexey G. Ryazanov, Rutgers University) and as expected by the Mendelian law, in the first generation we obtained about 50% of the mice with the genotype Scn1a+/+ eEF2K ± and 50% with the genotype Scn1a ± eEF2K ± . We then crossed the Scn1a ± eEF2K ± mice in order to obtained the three main genotypes needed for our studies: Scn1a+/+ eEF2K+/+ (called WT mice), Scn1a ± eEF2K+/+ mice (Scn1a ± mice) and Scn1a ± eEF2K−/− mice (Additional file 1: Figure S 2A). We also obtained Scn1a+/+ eEF2K−/− mice (eEF2K−/− mice) that were used to investigate if eEF2K deletion caused any behavioral alterations. To avoid genetic background influence, described in the Scn1a ± mice [42, 43], the mice were backcrossed for about 20 generations before were used for all the experiments described in the presented manuscript in order to obtain the same genetic background among genotypes. As expected the level of P-eEF2 was completely abolished in the eEF2K−/− and Scn1a ± eEF2K−/− mice (Additional file 1: Figure S 2B).

### eEF2K deletion modulates the expression of a set of proteins that regulate GABAergic synaptic transmission and the level of Akt phosphorylation

We previously showed that in eEF2K−/− mice the increased efficiency of GABAergic transmission was mediated by higher levels of expression of Synapsin 2b (Syn2b) in cerebral cortex and hippocampus and α5 subunit containing GABA_A_ receptor (GABA_A_Rα5) in hippocampus [18]. We thus analyzed the expression of Syn2b and GABA_A_Rα5 subunit in Scn1a ± and Scn1a ± eEF2K−/− mice and we confirmed a significant increased expression of both Syn2b and GABA_A_Rα5 in hippocampus and of Syn2b in cortex in mice deleted of eEF2K (eEF2K−/− and Scn1a ± eEF2K−/− mice). On the contrary, the expression of both Syn2b and GABA_A_Rα5 was not changed in the Scn1a ± mice (Fig. 1c, d). All these data suggest that deletion of eEF2K was able to strength inhibitory synapses in Scn1a ± mice.

Moreover, alteration of the Akt signaling has been also recently described in the Scn1a ± mice [44]. Interestingly our results showed that the increased levels of P-Akt in hippocampus and cortex of Scn1a ± mice were rescued to level similar to WT mice in the Scn1a ± mice by the deletion of eEF2K (Additional file 1: Figure S 3).

### eEF2K deletion protects Dravet mice from epileptic seizures onset

**Fig. 2:**
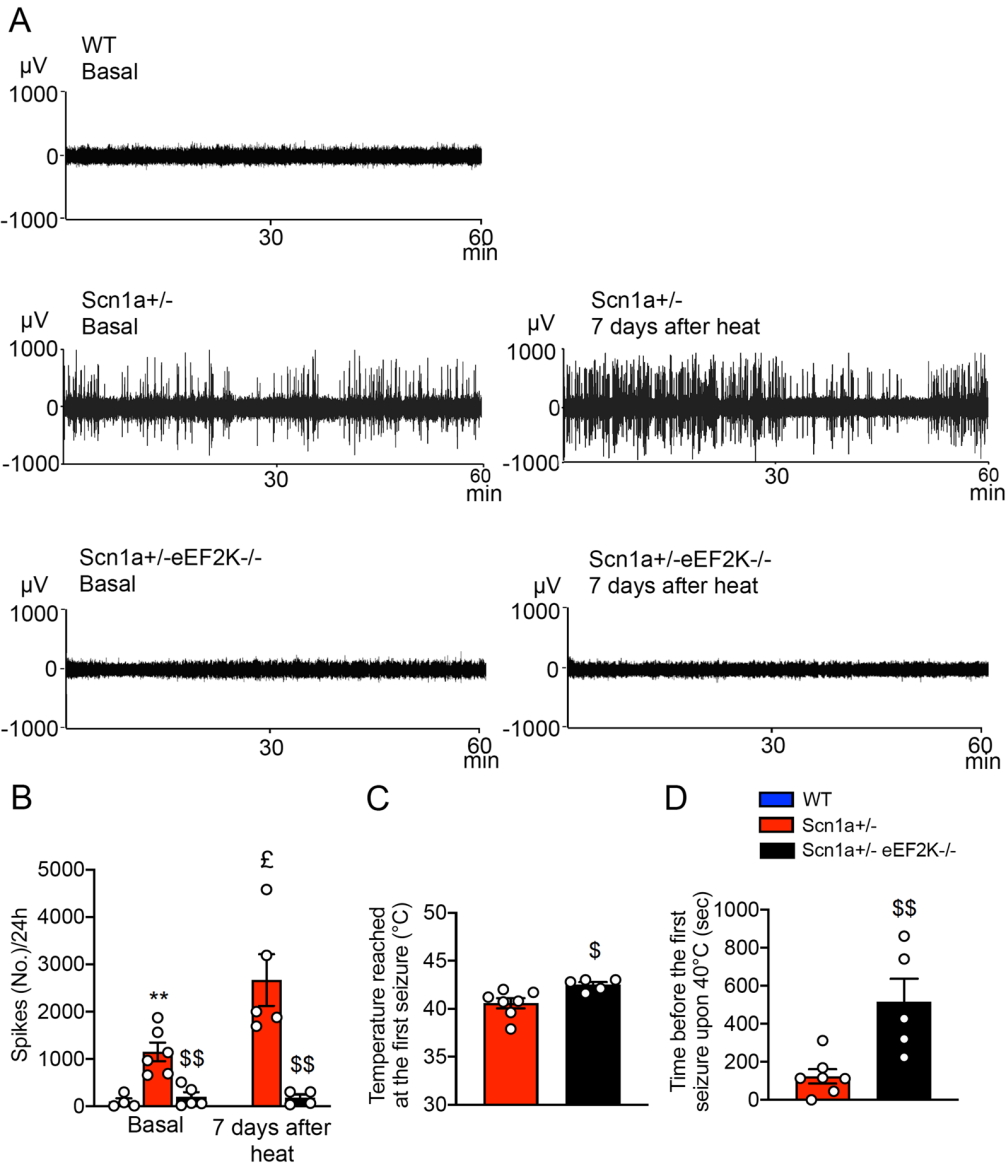
eEF2K deletion protects Scn1a ± mice from epileptic seizure onset.** A** Representative EEG traces (a representative 60 min. registration is shown) of a WT, Scn1a ± and Scn1a ± eEF2K−/− mice in basal condition (left) and 7 days after thermal stress (right). Scn1a ± mice show high number of EEG spikes in basal and 7 days after thermal stress condition when compared with WT and Scn1a ± eEF2K−/− mice. **B** Quantification of the number of EEG spikes per 24 h in basal condition (WT n = 4, Scn1a ± n = 6 and Scn1a ± eEF2K−/− n = 5) and 7 days after heat (Scn1a ± n = 5 and Scn1a ± eEF2K−/− n = 4). Scn1a ± eEF2K−/− mice are clearly less susceptible to seizure in basal and 7 days after thermal stress condition than Scn1a ± mice. Data are presented as mean ± SEM. Statistical analysis for number of EEG spikes in basal condition **p < 0.01 versus corresponding WT, $$*p <* 0.01 versus corresponding Scn1a ± ; One-way ANOVA test, Tukey’s post hoc. Statistical analysis for number of EEG spikes 7 days after thermal stress condition $$*p <* 0.01 versus corresponding Scn1a ± ; Unpaired two-tailed *t*-test. Statistical analysis £*p <* 0.05 versus corresponding Scn1a ± in basal condition; Unpaired two-tailed *t*-test. **C** Temperature reached at the first seizure. eEF2K deletion increase the minimum temperature necessary to reach the first seizure in Scn1a ± mice. Data are presented as mean ± SEM. Scn1a ± n = 7, Scn1a ± eEF2K−/− n = 5. Statistical analysis $*p <* 0.05 versus corresponding Scn1a ± ; Unpaired two-tailed *t*-test. **D** Time before the first seizure occur at 40 °C. eEF2K deletion increase the time before the first seizure occurred or until 42.5 °C was reached. Data are presented as mean ± SEM. Scn1a ± n = 7, Scn1a ± eEF2K−/− n = 5. Statistical analysis $$*p <* 0.01 versus corresponding Scn1a ± ; Unpaired two-tailed *t*-test

We first investigate the outcome of eEF2K deficiency on epileptic symptoms displayed by the Scn1a ± mice. We recorded electroencephalographic (EEG) traces from mice for 24 h and we counted the number of spikes that Scn1a ± mice and Scn1a ± eEF2K−/− mice displayed compared to WT mice. While Scn1a ± mice displayed an increased number of spikes in basal condition compared to WT mice, the Scn1a ± eEF2K−/− mice showed an EEG trace not different from WT mice, suggesting that eEF2K depletion was able to rescue the altered EEG observed in the Scn1a ± mice (Fig. 2a left panels, b). In Dravet syndrome, the onset of the epileptic seizures is triggered by an episode of fever and, usually, the following seizures arise without thermal stimuli [8]. This feature is maintained in Scn1a ± mice [26], therefore we triggered the convulsions by increasing mice body temperature and we counted the number of spikes 7 days after. We found a strong increase of number of spikes measured in 24 h in Scn1a ± mice after the thermal stress but not in the Scn1a ± eEF2K−/− mice, suggesting that eEF2K deficiency reduced the susceptibility to epilepsy of the Scn1a ± mice (Fig. 2a right panels, b). Furthermore, eEF2K depletion significantly increased the minimum temperature necessary to trigger epileptic seizures (Fig. 2c) and the time before the first seizure appear upon the body temperature reach 40 °C in comparison to Scn1a ± mice (Fig. 2d).

We also measured the number of epileptic episodes (epileptic discharges) occurring in 24 h in WT, Scn1a ± and Scn1a ± eEF2K−/− mice and in Scn1a ± and Scn1a ± eEF2K−/− 7 days after inducing convulsions by increasing mice body temperature. We found that the number of epileptic episodes was almost completely abolished in the Scn1a ± eEF2K−/− mice (Additional file 1: Figure S 4A and B).

### eEF2K deletion enhances GABAergic transmission in Scn1a ± mice

**Fig. 3:**
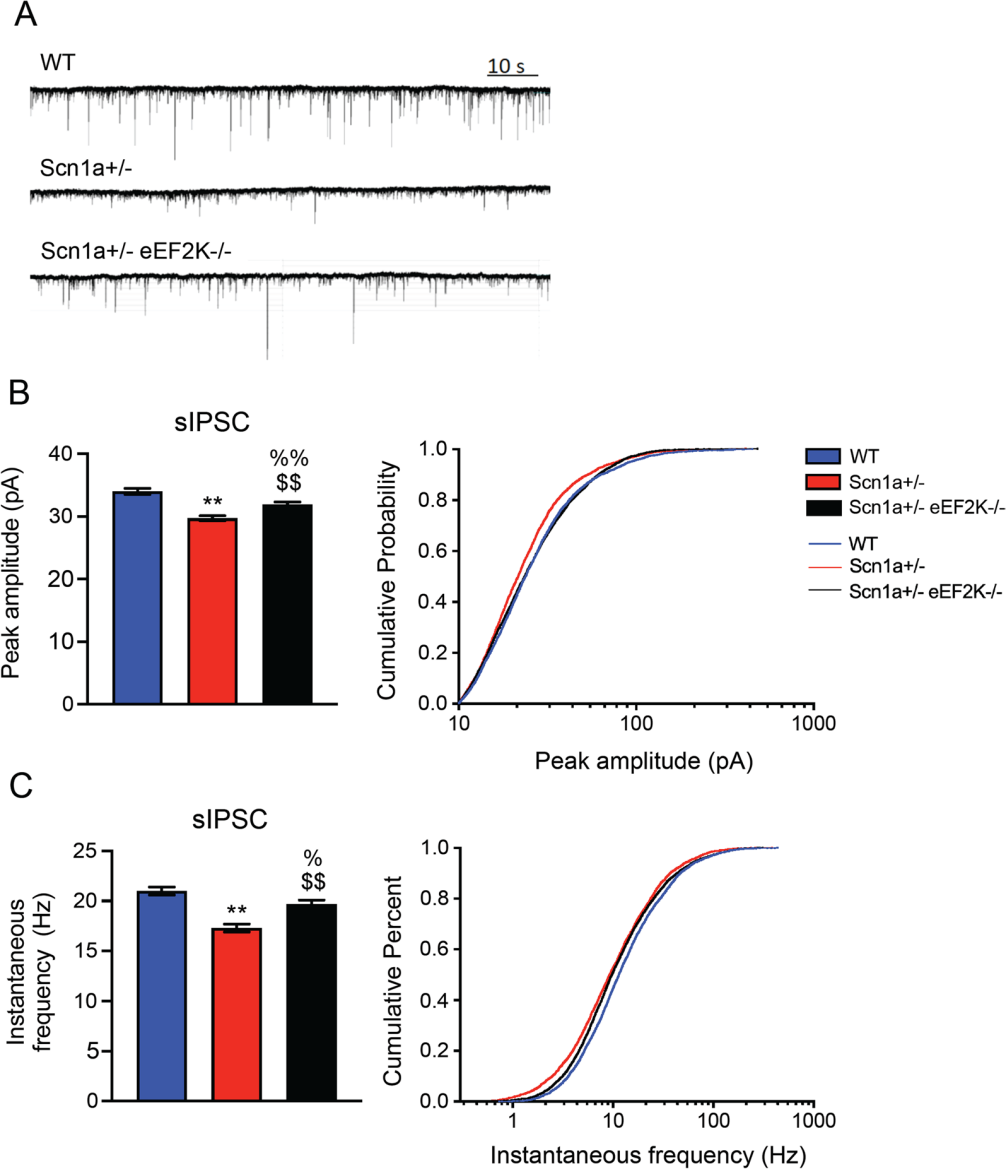
eEF2K deletion enhances GABAergic transmission in Scn1a ± mice.** A** Representative sIPSC traces measured in hippocampal neurons of CA1 region of WT, Scn1a ± and Scn1a ± eEF2K−/− mice. **B,C** Quantification of peak amplitude **(B)** and instantaneous frequency **(C)** from sIPSC traces. All data are presented as mean ± SEM. Statistical analysis for peak amplitude **p < 0.001, %%p < 0.05 versus corresponding WT; $$p < 0.001 versus Scn1a ± ; One-way ANOVA, Bonferroni. Statistical analysis for instantaneous frequency **p < 0.001, %*p <* 0.01 versus corresponding WT; $$p < 0.05 versus Scn1a ± ; One-way ANOVA, Bonferroni

A hyperexcitable neuronal network is one of the major causes of the epileptic seizures in Dravet syndrome [23, 45]. In our previous publication [18] we clearly showed that both frequency and amplitude of mEPSCs measured in granule cells of dentate gyrus of the eEF2K−/− mice where not changed compared to WT mice, while both frequency and amplitude of mIPSCs were increased in the eEF2K−/− mice compared to WT mice. Thus our previous data suggest that deletion of eEF2K reduces the E/I balance by increasing the GABAergic transmission. We thus focused our attention on measuring only inhibitory postsynaptic currents (sIPSC) because we believe that the excitatory synapses are not affected by the deletion of eEF2K. We recorded sIPSC in pyramidal neurons of the CA1 region of the hippocampus in brain slices from WT, Scn1a ± and Scn1a ± eEF2K−/− mice. As expected, Scn1a ± mice displayed reduced amplitude and instantaneous frequency in the recorded sIPSCs compared to WT mice [13]. Interestingly eEF2K depletion enhanced both amplitude and instantaneous frequency of sIPSC of Scn1a ± mice. However, the rescue was partial as the IPSC amplitude and instantaneous frequency measured in Scn1a ± eEF2K−/− were significantly smaller compared to WT mice (Fig. 3a–c).

### eEF2K deletion ameliorates motor coordination in Scn1a ± mice

Motor deficits are common in Dravet syndrome: most of the patient are affected by ataxia, dysarthria, intention tremor and eye movement disorder [6] and these features are replicated in Scn1a ± mice [13, 19]. We thus tested WT, Scn1a ± and Scn1a ± eEF2K−/− mice for motor function with the spontaneous motor activity test, the limb force with the wire hanging test and the motor coordination with balance beam, pole test and rotarod test. As previously demonstrated by Heise et al., eEF2K−/− does not show alteration in motor function and limb force when compared with WT mice [18]. First, we verified the effect of eEF2K deletion on motor coordination comparing eEF2K−/− mice with WT mice. As showed in the Additional file 1: Figure S 5 motor coordination were also not altered in the eEF2K−/− mice (Additional file 1: Figure S 5A–C).

**Fig. 4:**
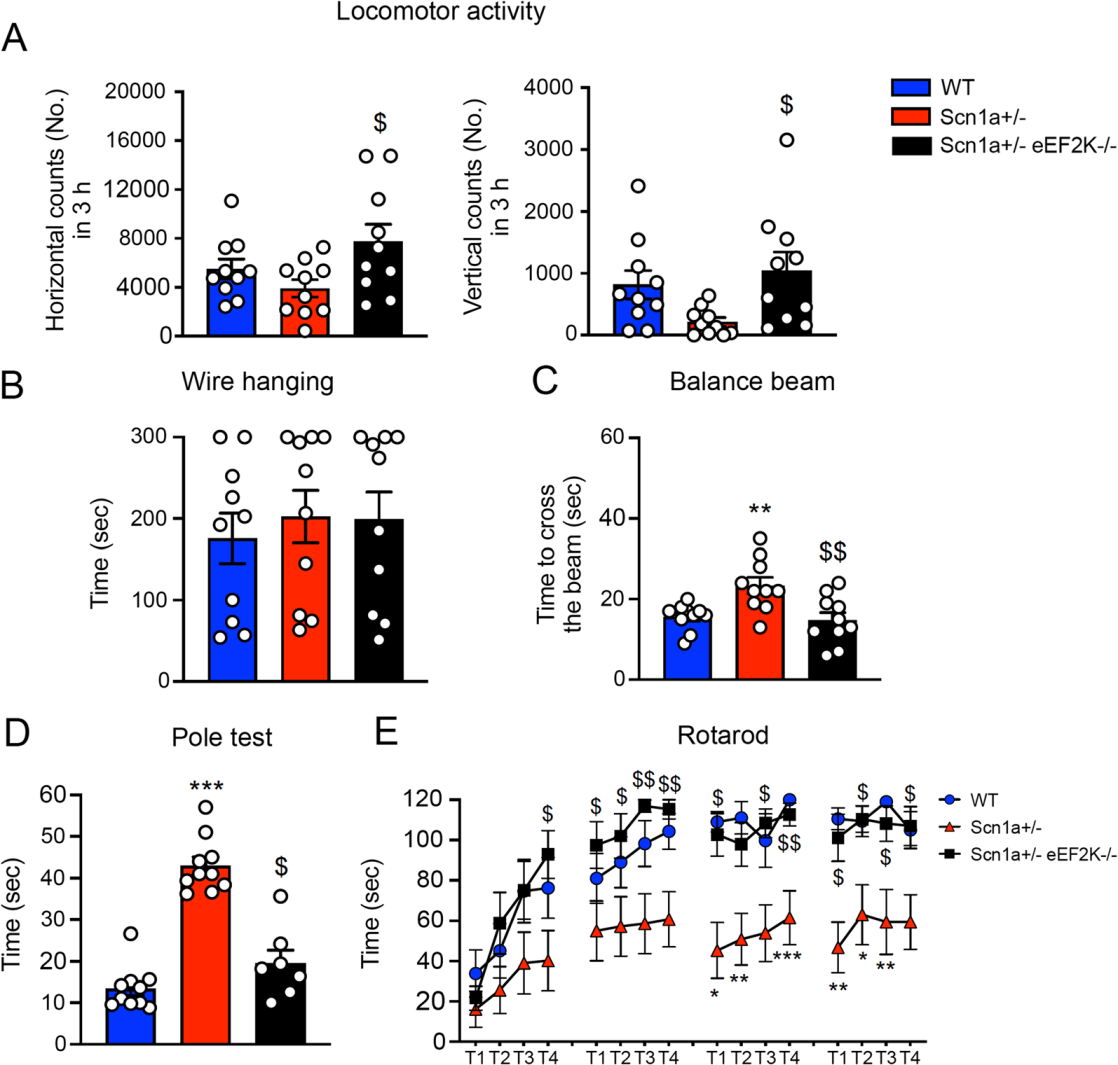
eEF2K deletion rescues balance impairment and motor coordination in Scn1a ± mice.** A** Counting of horizontal (left) and vertical (right) movements occurred in 3 h in the locomotor activity test. Scn1a ± eEF2K−/− mice show high number of horizontal and vertical movements. Scn1a ± had no difficulty in movement or climbing. Data are presented as mean ± SEM. N = 10 per groups. Statistical analysis $*p <* 0.05 versus corresponding Scn1a ± ; One-way ANOVA, Tukey’s post hoc. **B** Time to fall in wire hanging test measured over 300 s. Data are presented as mean ± SEM. N = 10 per groups. One way-ANOVA, Tukey’s post hoc. **C** Time to cross the 12 mm beam of trained WT, Scn1a ± and Scn1a ± eEF2K−/− mice. Scn1a ± mice are impaired in the balance beam test when compared with WT and Scn1a ± eEF2K−/− mice. All data are presented as mean ± SEM. N = 10 per groups. Statistical analysis **p < 0.01 versus corresponding WT, $$*p <* 0.01 versus corresponding Scn1a ± ; One way-ANOVA, Tukey’s post hoc. **D** Time to downstream the pole in pole test. Scn1a ± mice are impaired in the pole test when compared with WT and Scn1a ± eEF2K−/− mice. All data are presented as mean ± SEM. WT n = 10, Scn1a ± n = 10, Scn1a ± eEF2K−/− n = 7. Statistical analysis ***p < 0.001 versus corresponding WT; $*p <* 0.05 versus Scn1a ± ; Kruskal–Wallis test, Dunn’s post hoc. **E** Scn1a ± mice show impaired motor coordination in rotarod test when compared with WT and Scn1a ± eEF2K−/− mice. The plot shows mean ± SEM latency to fall from an accelerating rotarod during trials. N = 10 per group. Statistical analysis *p < 0.05, **p < 0.01, ***p < 0.001 versus corresponding WT; $*p <* 0.05, $$*p <* 0.01 versus Scn1a ± ; Kruskal–Wallis test, Dunn’s post hoc

WT and Scn1a ± mice were then compared to the Scn1a ± eEF2K−/− mice. Movements in horizontal and vertical directions were not statistically different, meaning that Scn1a ± mice had no difficult in walking or climbing. Also Scn1a ± eEF2K−/− mice did not display any difficulties in movements, even if both horizontal and vertical movements were slightly significantly higher compared to Scn1a ± mice (Fig. 4a). We also measure the latency to fall in wire hanging test as measure of limb muscle strength. We did not fin any difference in terms of muscle strength among WT, Scn1a ± and Scn1a ± eEF2K−/− mice (Fig. 4b).

We then assayed the motor coordination with balance beam and we found that, while the Scn1a ± mice took longer time compare WT mice to cross the 12 mm beam, the Scn1a ± eEF2K−/− mice behaved as the WT mice (Fig. 4c). With the pole test we measured the latency to turn downwards and descend and we observed that the Scn1a ± mice took longer time than WT mice while the Scn1a ± eEF2K−/− mice behaved as WT mice (Fig. 4d). Finally, in the rotarod test the Scn1a ± mice showed a strong impairment in motor coordination that was rescued in the Scn1a ± eEF2K−/− mice (Fig. 4e). All these data clearly demonstrated that deletion of eEF2K was able to recover all the motor deficits found in the Scn1a ± mice.

### eEF2K deletion rescues the stereotyped behavior of Scn1a ± mice

Dravet syndrome patients are affected also by important and diverse patient-related co-morbidity such as stereotyped behaviors and anxiety.

**Fig. 5:**
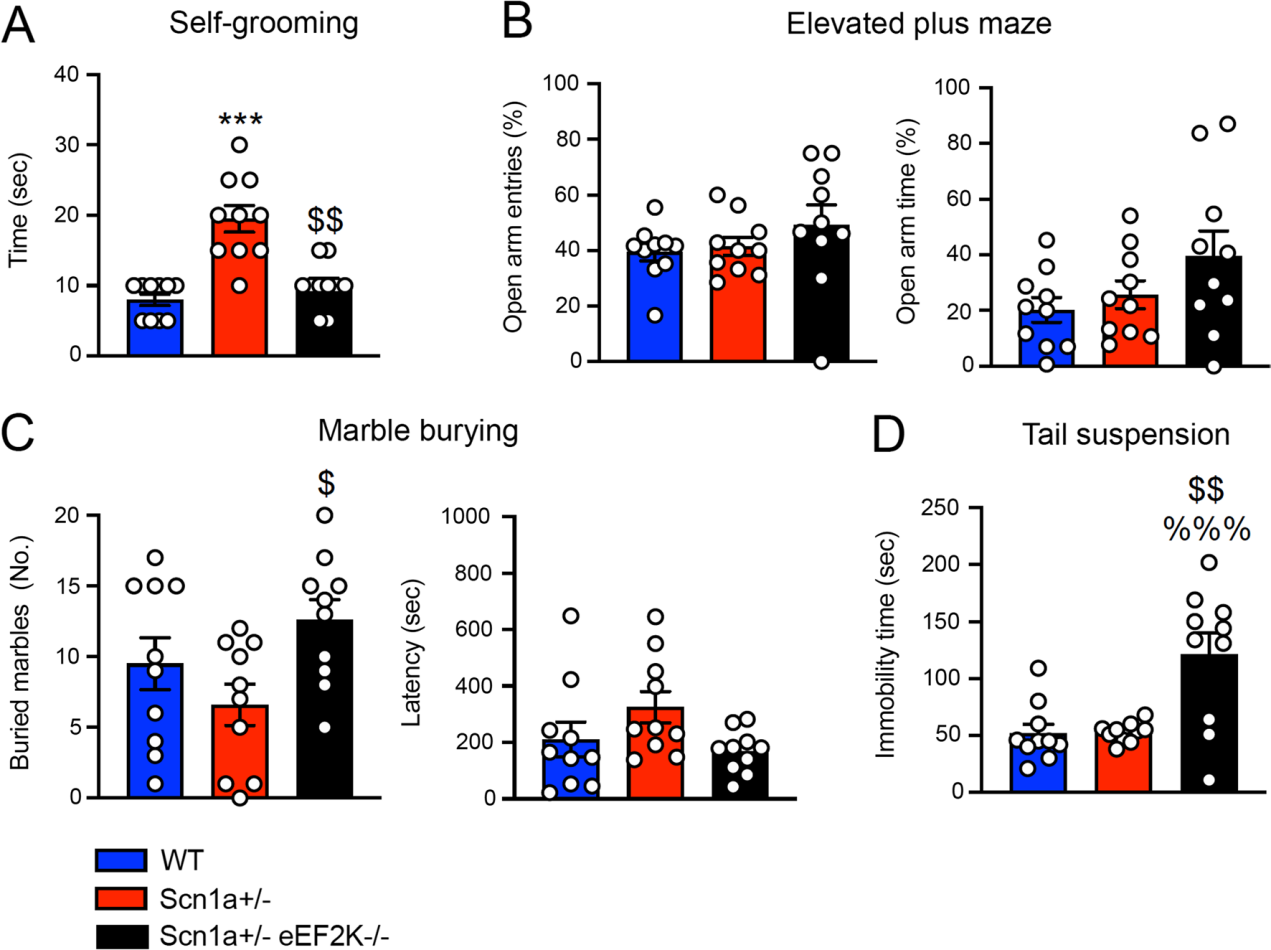
Genetic deletion of eEF2K rescues autistic-like behavior in Scn1a ± mice.**A** Significantly increase in repetitive grooming behavior in Scn1a ± mice compared with WT and Scn1a ± eEF2K−/− mice evaluated in terms of time spent in self-grooming over 10 min observation. Data are presented as mean ± SEM. N = 10 per group. Statistical analysis ***p < 0.001 versus corresponding WT; $$*p <* 0.01 versus corresponding Scn1a ± ; Kruskal–Wallis test, Dunn’s post hoc. **B,C** Scn1a ± mice do not show anxiety-behavior in elevated plus maze**(B)** and marble burying **(C)** test. **B** Open arm entries (left) and time spend in open arms (right), evaluated in the elevated plus maze test over 5 min. Data are presented as mean ± SEM. N = 10 per group. One-way ANOVA, Tukey’s post hoc. **C** Number of marbles buried (left) and latency to the first buried (right) evaluated in the marble-burying test over 15 min. Data are presented as mean ± SEM. N = 10 per group. Statistical analysis $*p <* 0.05 versus corresponding Scn1a ± ; One-way ANOVA, Tukey’s post hoc. **D** Time spent immobile in tail suspension test do not show depressive-like behaviors in Scn1a ± mice when compared with WT mice. Scn1a ± eEF2K−/− mice show depressive-like behavior when compared with WT and Scn1a ± mice. Data are presented as mean ± SEM. N = 10 per group. Statistical analysis %%%*p <* 0.001 versus corresponding WT, $$*p <* 0.01 versus corresponding Scn1a ± ; One-way ANOVA, Tukey’s post hoc

We evaluated stereotyped behaviors by measuring the time spent in self-grooming. We observed that EF2K deletion in Scn1a ± mice was able to reduce the time occupied in self-grooming of Scn1a ± mice to a level similar to the WT mice (Fig. 5a). As expected the eEF2K−/− mice did not show any increased grooming activity compare to WT mice (Additional file 1: Figure S 6A).

Then we tested mice for anxiety-like behavior. We did not found difference among the three genotypes nor in the elevated plus maze nor in the marble burying test demonstrating that eEF2K deletion did not affect anxiety-like behavior (Fig. 5b, c), as also previously demonstrated by Heise et al. where eEF2K−/− mice behave like WT when tested for anxiety-like behavior [18]. On the contrary when we analyzed depression-like behavior by using the tail suspension test, we found that eEF2K deletion was associated to an increased immobility time in the tail suspension test, both when deleted in the WT mice (Additional file 1: Figure S 6B) and in the Scn1a ± mice (Fig. 5d).

### eEF2K deficiency ameliorates episodic and spatial memory and social novelty preference of Scn1a ± mice

**Fig. 6:**
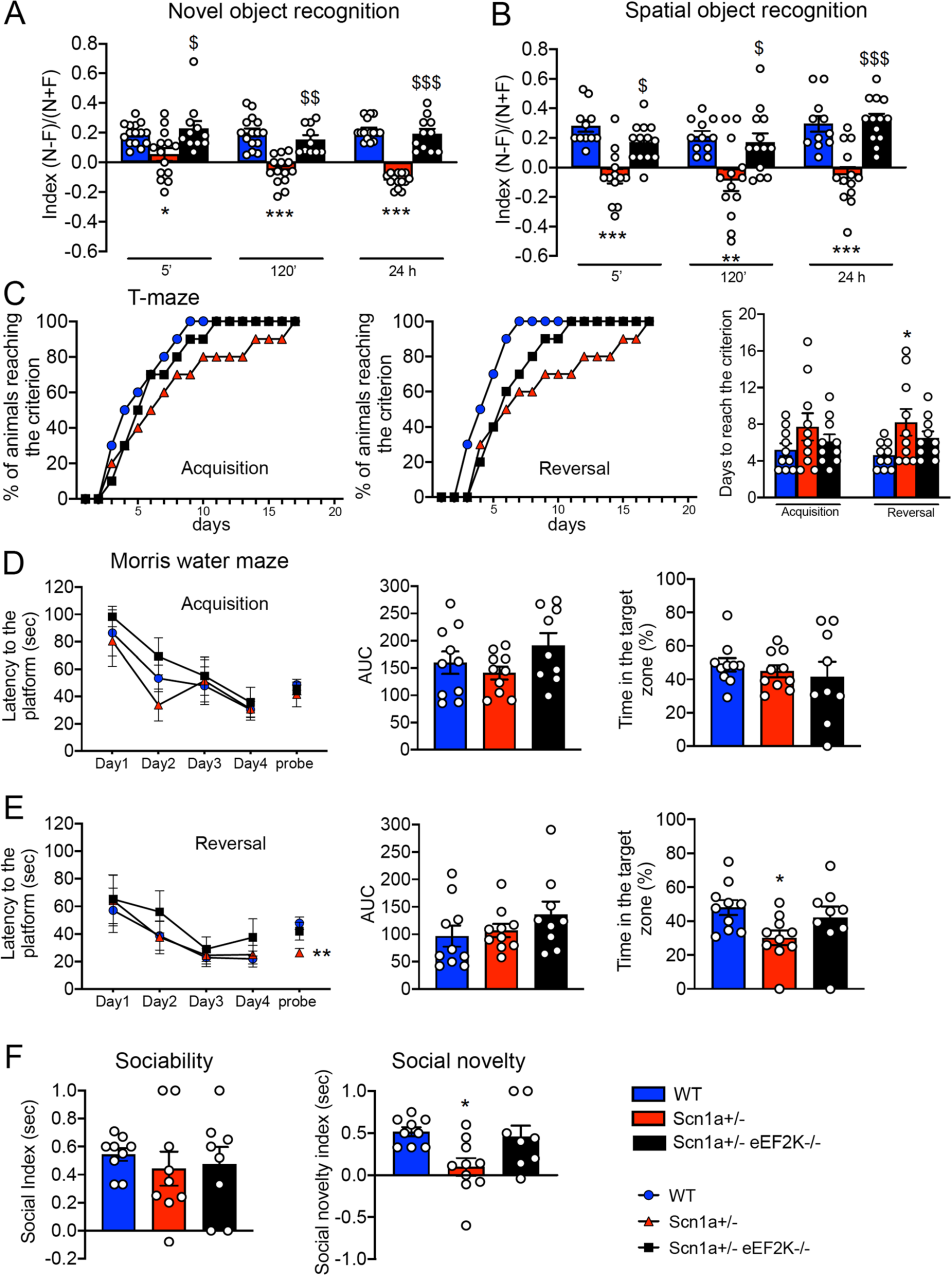
eEF2K deletion in Scn1a ± mice rescues the learning and memory impairment and social recognition alteration.** A, B** Discrimination index evaluated in the novel object recognition test **(A)** and in the spatial object recognition test **(B)**. **A** Scn1a ± mice show an impairment in novel object recognition at 5 min, 120 min and 24 h after the familiarization phase compared with WT and Scn1a ± eEF2K−/− mice. Data are presented as mean ± SEM. 5 min and 24 h: WT n = 15, Scn1a ± n = 14, Scn1a ± eEF2K−/− n = 11; 120 min WT n = 15 Scn1a ± n = 14, Scn1a ± eEF2K−/− = 10. Statistical analysis *p < 0.05, ***p < 0.001 versus corresponding WT, $*p <* 0.05, $$*p <* 0.01, $$$*p <* 0.001 versus corresponding Scn1a ± ; Kruskal–Wallis test, Dunn’s post hoc. **B** Scn1a ± mice are impaired in spatial object recognition test at 5 min, 120 min and 24 h after familiarization phase when compared with WT and Scn1a ± eEF2K−/− mice. Data are presented as mean ± SEM. WT n = 11, Scn1a ± n = 13, Scn1a ± eEF2K−/− n = 13. Statistical analysis **p < 0.01, ***p < 0.001 versus corresponding WT, $*p <* 0.05, $$$*p <* 0.001 versus corresponding Scn1a ± ; Kruskal–Wallis test, Dunn’s post hoc for 5 min and One-way ANOVA, Tukey’s post hoc for 120 min and 24 h. **C** Scn1a ± mice required more days to achieve the criterion than WT mice during the reversal phase of T-maze. Data are presented as mean ± SEM. N = 10 per group. Statistical analysis *p < 0.05 versus corresponding WT. Days to reach the criterion was analyzed by One-way ANOVA, Tukey’s post hoc. The percentage of animals reaching the criterion was analyzed by Fisher's exact test. **D, E** Acquisition and reversal learning in Morris water maze task in terms of latency to reach the platform across 4 days trial and probe test and in percentage of the time spent in the target quadrant during the probe test. **D** Scn1a ± mice display normal Morris water maze learning in the acquisition phase in both latency to reach the platform and in probe test. Data are presented as mean ± SEM. WT, Scn1a ± n = 10, Scn1a ± eEF2K−/− n = 9. Each day latency was analyzed by Kruskal–Wallis test, Dunn’s post hoc and the acquisition probe test latency was analyzed by One-way ANOVA, Tukey’s post hoc. AUC and percentage in the target zone were analyzed by One-way ANOVA, Tukey’s post hoc. **E** Scn1a ± mice show an impaired spatial memory in Morris water maze probe test in the reversal phase. Data are presented as mean ± SEM. WT, Scn1a ± n = 10, Scn1a ± eEF2K−/− n = 9. Each day of latency was analyzed by Kruskal–Wallis test, Dunn’s post hoc. Statistical analysis in reversal probe test latency **p < 0.01 versus corresponding WT; One-way ANOVA, Tukey’s post hoc. AUC was analyzed by Kruskal–Wallis test, Dunn’s post hoc. Statistical analysis for percentage of time in the target zone *p < 0.05 versus corresponding WT; One-way ANOVA, Tukey’s post hoc. **F** Sociability index (left) and social novelty preference index (right) evaluated in sociability (left) and in social novelty test (right). Scn1a ± mice display a normal social attitude when compared with WT and Scn1a ± eEF2K−/− mice in sociability test (left). Data are presented as mean ± SEM. WT, Scn1a ± n = 9, Scn1a ± eEF2K−/− n = 8; One-way ANOVA, Tukey’s post hoc. Scn1a ± display an impairment in social recognition when tested for social novelty test (right). Data are presented as mean ± SEM. WT n = 9, Scn1a ± n = 10, Scn1a ± eEF2K−/− n = 8. Statistical analysis *p < 0.05 versus corresponding WT; One-way ANOVA, Tukey’s post hoc

Another typical trait of Dravet patients is the presence of cognitive impairments [3, 8]. We tested mice for episodic and spatial memory using novel object recognition test and spatial object recognition test, respectively. Mice had to recognize the unfamiliar or displaced object after 5 min, 120 min or 24 h from familiarization phase. In the novel object recognition test Scn1a ± mice were unable to recognize the new object as unfamiliar, as shown by the reduced discrimination index at all the time points compared to WT mice, suggesting that Scn1a ± mice had deficits in episodic memory. Interestingly the Scn1a ± eEF2K−/− mice, as well as WT mice, displayed a strong preference for the unfamiliar object, implicating that eEF2K deletion had a beneficial effect on Scn1a ± mice episodic memory (Fig. 6a). Compared to WT mice the eEF2K−/− mice did not showed any impairment (Additional file 1: Figure  S 6C and D).

In the spatial object recognition test, Scn1a ± mice displayed a defect preferring the stationary object, as shown by the negative discrimination index. On the contrary Scn1a ± eEF2K−/− performed in the test similarly to the WT mice spending more time exploring the displaced object (Fig. 6b) confirming the positive effect of eEF2K deletion on memory performances.

In the Morris water maze and T-maze tests, two other memory related tests, Scn1a ± mice performed as the WT mice in the acquisition phase (Fig. 6c left and right, d) while showed a significant impairment in the reversal phase of the test (Fig. 6c center and right, e) that was ameliorated following the deletion of eEF2K (Fig. 6c right, e).

We then analyzed the social behavior of WT, Scn1a ± and Scn1a ± eEF2K−/− mice using the sociability and social novelty preference test. Scn1a ± mice exhibited more interest for the unfamiliar mouse than for the empty cage, as well as WT and Scn1a ± eEF2K−/− mice, demonstrating a normal attitude in social interaction. However, in the social novelty test, Scn1a ± mice, differentially to WT mice, spent the same time in exploring familiar and unfamiliar mice, indicating impairment in the social recognition. Interestingly the deletion of eEF2K in the Scn1a ± was able to rescue this behavioral alteration (Fig. 6f). Indeed the eEF2K−/− mice were not different from WT mice for both social novelty test (Additional file 1: Figure S 6E) and sociability [18].

Finally we also observed that Scn1a ± mice, showed some impairment in visual cliff tests, but not in the olfactory test, and that eEF2K deletion was able to rescue this behavioral alteration (Additional file 1: Figure  S 7A and B).

### Pharmacological rescue of EEG alterations of Scn1a ± mice using an eEF2K inhibitor

We demonstrated that the genetic deletion of eEF2K in the Scn1a ± mice rescued the main phenotypes such as epileptic seizure and deficit in behavior, which characterized the Dravet syndrome suggesting eEF2K as a possible pharmacological target.

To confirm our hypothesis, we tested the ability of A484954 of reverting EEG alterations found in Scn1a ± mice. A484954 is a selective small molecule inhibitor for eEF2K that inhibits eEF2K activity in an ATP-competitive but not CaM-dependent manner [46], resulting in an inhibition of eEF2 phosphorylation at Thr 56. To delivery A484954 in mice we used the pellet implantation methodology provided by Innovative Research of America company (IRA), instead of classical intraperitoneal injection. The pellets, loaded with placebo or A484954, are able to release the same quantity of drug or placebo each day for 5 or 10 days, depending on the size and composition of the pellets.

**Fig. 7:**
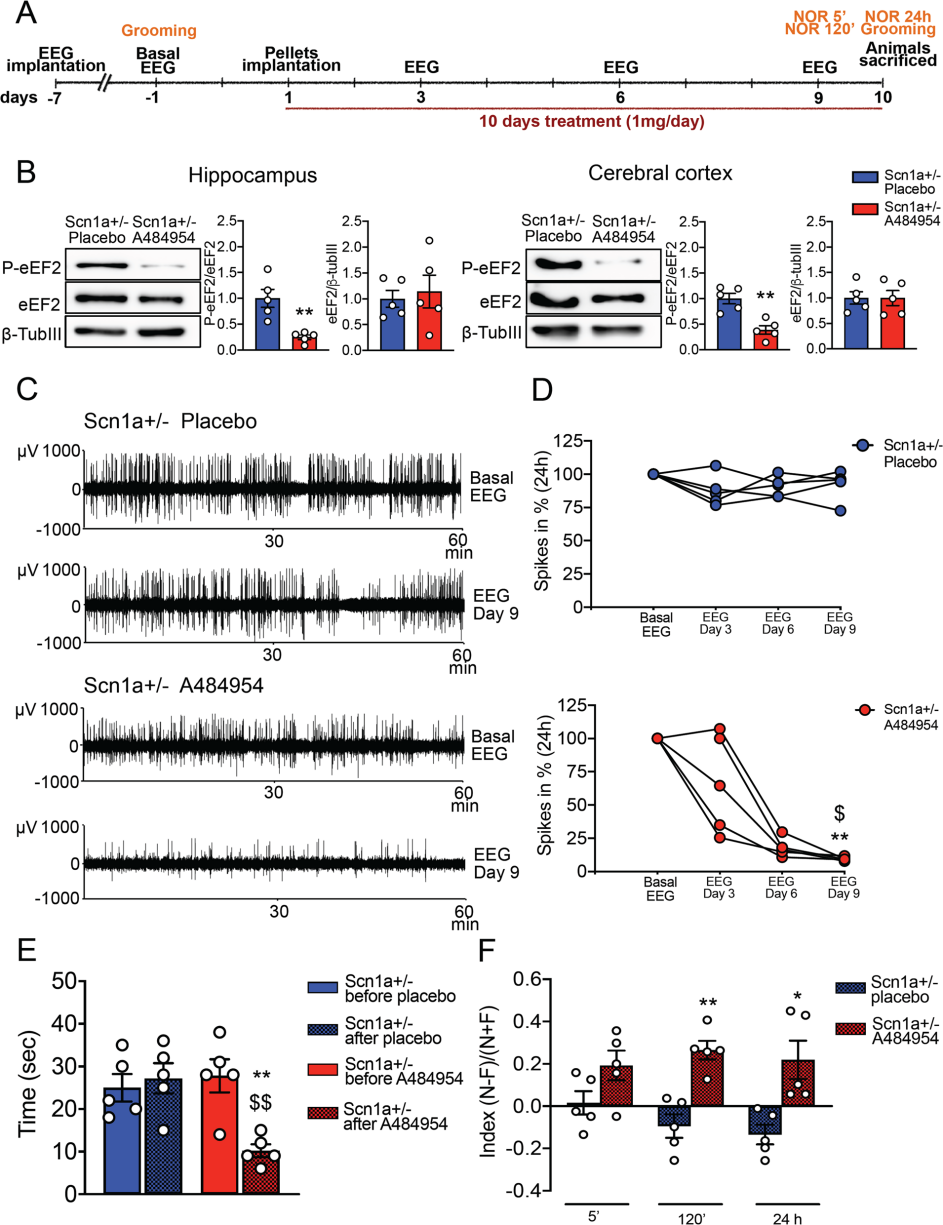
Pharmacological inhibition of eEF2K activity rescues the epileptic phenotype in Scn1a ± mice and improve autistic-like behavior and memory impairment.**A** Experimental timeline. **B** Representative western blots and relative quantification show a significantly reduction of phosphorylated eEF2 and no differences in eEF2 expression levels in total homogenate of hippocampus (left) and cerebral cortex (right) of Scn1a ± mice after a chronic treatment of 10 days with 1 mg/day dose of A484954 compared with placebo. All data are presented as mean ± SEM. N = 5 per group. Statistical analysis **p < 0.01 versus corresponding Scn1a ± treated with placebo; Unpaired two-tailed *t*-test. **C** Representative EEG traces (a 60 min registration is shown) in basal condition and at 9 days of treatment of a Scn1a ± mouse treated with placebo or A484954. **D** Quantification of the number of EEG spikes calculated in percentage per 24 h in basal condition and at 3, 6 and 9 days of treatment. Significant reduction of the number of EEG spikes across the three EEG recording in Scn1a ± mice treated with A484954. No difference in number of EEG spikes in Scn1a ± mice treated with placebo. Scn1a ± mice treated with A484954 n = 5, Scn1a ± mice treated with placebo n = 5. Statistical analysis **p < 0.01 versus corresponding EEG basal, $*p <* 0.05 versus corresponding EEG at 3 days of treatment; Friedman test. **E** Significant reduction in repetitive grooming behavior in Scn1a ± mice before and after treatment with A484954 and in Scn1a ± mice treated with A484954 compared with Scn1a ± mice treated with placebo. Repetitive grooming is evaluated as time spent in self-grooming over 10 min observations. Data are presented as mean ± SEM. N = 5 per group. Statistical analysis **p < 0.01 versus corresponding Scn1a ± mice before treatment with A484954; $$*p <* 0.01 versus corresponding Scn1a ± mice after treatment with placebo; Two-way ANOVA. **F** Discrimination index evaluated in the novel object recognition test at the end of treatment shows a memory improvement in Scn1a ± mice treated with A484954 compared with animals treated with placebo at 120 min and 24 h after familiarization phase. Data are presented as mean ± SEM. N = 5 per group. Statistical analysis *p < 0.05, **p < 0.01 versus corresponding Scn1a ± mice treated with placebo. Unpaired two-tailed *t*-test for 5 min and 120 min; Two-tailed Mann–Whitney test for 24 h

Mice were implanted with the pellets on the lateral side of the neck between the ear and the shoulder. We first identify the minimal doses able to almost completely abolish the phosphorylation of eEF2 in WT mice. We tested pellets able to release 0.5 mg/day or 1 mg/day for 5 days, and 0.5 mg/day or 1 mg/day for 10 days. We found that the only dose able to almost completely abolish the phosphorylation of eEF2 in cerebral cortex, hippocampus, liver, kidney and heart was 1 mg/day for 10 days (Additional file 1: Figure S 8). Placebo pellets were used in the experiment as the proper control. The treatment was performed as described in Fig. 7a. First, we confirmed a significant reduced phosphorylation of eEF2 in the hippocampus and cerebral cortex of Scn1a ± mice treated with A484954 in comparison with the ones treated with placebo after 10 days of treatment, confirming that A484954 was able to inhibit eEF2K activity in brain of Scn1a ± mice (Fig. 7b).

We then measure the EEG number of spikes in the same mice before pellet implantation (EEG basal), and at 3, 6 and 9 days of treatment. We observed a progressive decrease of number of spikes in the Scn1a ± mice treated with A484954 (Fig. 7c, d) demonstrating that also pharmacological inhibition of eEF2K was able to strongly reduce the number of EEG spikes, and probably to ameliorate the epileptic phenotype, of the Scn1a ± mice.

We also found that the treatment with A484954 was able to strongly reduces grooming behavior and improve episodic memory performance measured with novel object recognition test. For the grooming test Scn1a ± mice were tested before and after drug treatment and only the mice treated with A484954 showed a significant reduction of the grooming activity (Fig. 7e). Mice treated for 10 days with A484954 displayed a strong preference for the unfamiliar objects, implicating that pharmacological inhibition of eEF2K deletion had a beneficial effect on Scn1a ± mice impaired episodic memory (Fig. 7f).

## Discussion

We have previously identified eEF2K, a kinase involved in the regulation of protein translation, as an effective modulator of GABAergic synaptic transmission [18]. Indeed, we demonstrated that genetic and pharmacological eEF2K inhibition rescued the epileptic phenotype in a genetic animal model of epilepsy, the Synapsin1 knock out mice [18]. This work provides strong evidence that genetic deletion of eEF2K in the Scn1a ± mice, a mouse model of Dravet syndrome, can rescue both epileptic phenotype and behavioral alterations.

The function of eEF2K in regulating synaptic plasticity and the excitation/inhibition balance in the brain has been widely studied using in vitro and in vivo models, suggesting that eEF2K dysregulation may contribute to the pathogenesis of a subset of neurodegenerative and neuropsychiatric disorders [47–50] and featuring the eEF2K/eEF2 pathway as a valuable pharmacological target for brain related disease treatment. High levels of phosphorylated eEF2 have been described in Alzheimer disease [51, 52], Parkinson disease [48] and genetic epilepsy [18] and it has also been reported that inhibition of eEF2K activity ameliorate the pathology [18, 47, 51, 52].

We found that phosphorylation of eEF2, the only substrate of eEF2K, is higher in the hippocampus and cerebral cortex of Scn1a ± mice compared with WT, while no difference was found in the liver, kidney and heart. We measured a higher level of phosphorylated eEF2 in adult Scn1a±mice (3 months old mice) when the high number of spikes and the behavioral deficit are clearly displayed. The increased level of eEF2 phosphorylation was maintained also in 9 months old mice.

We previously found a high level of P-eEF2 in the Synapsin 1 KO mice (Syn1-KO), another mouse model of genetic epilepsy. At the current state of art the underlying mechanism for both genetic models is still unknown, although we can speculate some possible mechanisms. High levels of P-eEF2 were reported in a mouse model of Alzheimer’s disease [47] and in brain samples of patients affected by Alzheimer’s disease [47, 53] as a consequence of the pathological activation of AMPK followed by the activation of eEF2K [51]. Possibly, a similar mechanism might be evoked in the Scn1a ± mice in which the overexcitation could generate a vicious cycle of over-increasing network activity. Chronically increased neuronal excitation might lead to the excitatory synapse dependent activation of the eEF2K/eEF2 pathway and the subsequent dampening of the GABAergic synapses would further increase network activity, thereby aggravating the epilepsies in the Scn1a ± mice.

Indeed, even if the molecular mechanism responsible for the increased level of eEF2 phosphorylation in the Scn1a ± mice remain to be determined, we demonstrated that the genetic deletion of eEF2K was sufficient to strongly reduce the interictal activity in basal and under thermal stress conditions.

As previously reported in the literature [13, 15], mutations in the Nav1.1 channel caused selective reduction in sodium currents and action potential firing of GABAergic interneurons in hippocampus neocortex, cerebellum and thalamus in Scn1a ± mice leading to reduced GABAergic transmission [13, 19, 45, 54, 55]. Given that eEF2K−/− mice exhibited a stronger GABAergic synaptic transmission and no change in the excitatory synaptic strength [18], we wondered whether this deficit in GABAergic transmission in Scn1a ± mice could be reverted by eEF2K deletion. We found that eEF2K deletion in Scn1a ± mice enhanced both amplitude and instantaneous frequency of sIPSCs in CA1 pyramidal neurons. Moreover, we demonstrated that eEF2K deletion specifically potentiated the synaptic and tonic GABAergic transmission increasing the expression of Synapsyn2b [56] and GABA_A_Rα5 [57, 58] respectively. In conclusion, these data suggest that eEF2K deletion is sufficient to rescue the excitation/inhibition unbalance responsible for the epileptic phenotype of Scn1a ± mice by selectively increasing GABAergic transmission efficiency.

It has also been demonstrated that the PI3K/Akt/mTOR pathway is overactivated in different ASD and epilepsy mouse models including the Scn1a ± mouse model [44, 59]. We found that in these mice the increased level of phosphorylated Akt was rescued by the deletion of eEF2K. Given that alteration in Akt pathway is known to be involved in the pathogenesis of neurodevelopmental disorders [60, 61], our data might suggest that the restored level of phosphorylated Akt might contribute to the rescue of the behavioral alterations observed in the Scn1a ± mice after eEF2K deletion.

We then investigated the effect of eEF2K deletion on the motor and behavioral deficits of Scn1a ± mice. We first demonstrated that eEF2K deletion per se did not cause any behavioral alteration. Our behavioral characterization demonstrated that eEF2K−/− mice behave like WT mice in motor coordinator tests and in cortex-, hippocampus- and amygdala-dependent behavioral paradigms presenting only a mild hippocampal-dependent phenotype in the context of fear conditioning [18]. Thus, we confirmed that Scn1a ± mice recapitulated the majority of comorbidity found in Dravet syndrome patients. In particular, they showed impairments in motor coordination, with no alteration in walking and climbing or in muscle strength. Moreover Scn1a ± mice compared to WT mice had a reduced memory for objects and their spatial relationships. These memories and learning problems were also confirmed by the T-maze and Morris water maze test where Scn1a ± mice performed worse than WT mice during the reversal phase of the test. Scn1a ± mice also presented stereotyped behaviors although they did not show anxiety-like or major depression-like behaviors. Notably, Scn1a ± mice did not show any alteration of social interaction as previously demonstrated [19, 62]. However a similar finding, lack of social deficit in the 3-chamber sociability test, was described in another Scn1a ± mice [63].

All the behavioral alterations were rescued by the deletion of the eEF2K gene strongly suggesting that the inhibition of the eEF2K/eEF2 pathway by increasing the strength of the GABAergic transmission was sufficient not only to rescue the epileptic phenotype of Scn1a ± mice but also to ameliorate the associated comorbidity.

Several publications [20, 64–66] have clearly demonstrated that eEF2 is the only substrate of eEF2K. For this reason we think that the major, if not the only, consequence of eEF2K knockout is the lack of phosphorylation of eEF2. Our extensive analysis of the behavior and electrophysiological analysis on the eEF2K−/− mice [18] (and this paper) suggest that deletion of eEF2K does not impair the major behavioral phenotype even if GABAergic transmission is potentiated in these mice. Recently Taha et al. [66] demonstrated that the general eEF2K-KO and targeted KO in dentate gyrus (DG) excitatory mature neurons induce the enhancement of neurogenesis and upregulation of neurogenesis-related proteins. The increased neurogenesis was correlated with improved performance in DG-dependent learning. We cannot exclude that this phenomena is occurring in Scn1a ± eEF2K−/− mice and that the increased neurogenesis in the DG might contribute to the rescue of some behavioral alteration of Scn1a ± mice.

These results and our previous study, demonstrating that genetic inhibition of eEF2K rescued the epileptic phenotype in a different model of genetic epilepsy [18], indicate eEF2K as a possible target for develop new pharmacological the treatment for epilepsy. Thus, we tested the effect of pharmacological inhibition of eEF2K on Scn1a ± mice. Our results demonstrate that subchronic treatment with A484954, a selective eEF2K inhibitor, was able to ameliorate the epileptic phenotype of Scn1a ± mice by reducing eEF2 phosphorylation in the hippocampus and cerebral cortex. Even though future studies focused on clarifying the activity of eEF2K inhibition on Dravet syndrome comorbidity are necessary, our data suggest eEF2K pharmacological inhibition as an innovative therapy for Dravet syndrome.

## Limitations

A major limitation in the presented study is that the molecular mechanism underlying the increased phosphorylation of eEF2 in the Scn1a ± mice was not investigated. The high P-eEF2 might depend on the over activation of eEF2K or by the inhibition of the phosphatases responsible for the dephosphorylation of eEF2. Whether either one of the two or both mechanisms are involved was not determined. A second limitation is that the molecular mechanism underlining the genetic inhibition of eEF2K/eEF2 pathway in rescuing epileptic and behavioral alterations in the Scn1a ± was not fully determined. Our biochemical and electrophysiological data suggest that the deletion of eEF2K might strength the inhibitory transmission in the Scn1a ± mice. However a more detailed electrophysiological analysis of the activity of the inhibitory and excitatory neurons is required to clarify how the deletion of eEF2K inhibits the epileptic phenotype in the Scn1a ± mice. Finally our pharmacological study has several limitations. We use the A484954 that is considered one of the most specific eEF2K inhibitor. We treated adult mice only for ten days with minimal doses able to almost completely inhibit the phosphorylation of eEF2K. We found that this treatment was able to rescue EEG alterations and some behavioral defects such as high grooming activity and novel object recognition impairments. However we do not know whether this pharmacological treatment can rescue also the reduced IPSC and other behavioral impairments present in the Scan1a ± mice. In particular we don't know if A484954 treatment is able to reduce the E/I balance by increasing the GABAergic transmission. Nevertheless we think that it will be important to implement a more accurate pharmacological study only when new, more potent and specific eEF2K inhibitor will be available.

## Conclusions

In summary, Scn1a ± mice exhibited increased levels of eEF2 phosphorylation that were associated with EEG alterations and a greater responsiveness to seizures accompanied by behavioral abnormalities including memory impairment, ASD like behavior and motor coordination problems. eEF2K deletion in Scn1a ± mice was sufficient to rescue all these impairments enhancing GABAergic transmission and modulating the PI3K/Akt/mTOR pathway. Despite eEF2k is an ubiquitous and conserved protein, it has only one substrate [65] and its deletion in WT mice caused only minor impairments suggesting that inhibiting its function should not elicit serious side effects. Notably, pharmacological inhibition of eEF2K in adult mice was also sufficient to normalize the EEG profile of Scn1a ± mice proposing eEF2K as a new pharmacological target not only for Dravet syndrome but also for several neuronal diseases in which eEF2K activity is altered [50].

## Supplementary Information

**Additional file 1: Figure S1**. No difference in eEF2 phosphorylation levels in total homogenate of liver, kidney, and heart between Scn1a ± and WT mice.** (A-C)** Western blots and relative quantification for phosphorylated eEF2 in samples of liver **(A)**, kidney **(B)** and heart **(C)** show no significant difference in 3, 6 and 9 months old in Scn1a ± mice compared with WT mice. All Data are presented as mean ± SEM; N = 4 per group. One-sample *t*-test.** Figure S2. **Genetic characterization and levels of phosphorylated eEF2 in WT, Scn1a ± , eEF2K−/− and Scn1a ± eEF2K−/− mice.** (A)** Representative PCR for SCN1A gene (left). WT Scn1a+/+ (WT) mice display single band of 300 bp, heterozygous Scn1a ± mice display one at 300 bp and the other ad 150 bp. Representative PCR for eEF2K gene (right). Length of the bands for WT and KO is at the same high at 1.2 kb. Two different PCR were performed for WT and KO. **(B)** Representative western blot and relative quantification for phosphorylated eEF2 in hippocampus and cerebral cortex of eEF2K−/−, Scn1a ± , WT (Scn1a+/+ eEF2K+/ +) and Scn1a ± eEF2K−/− show that eEF2 phosphorylation is totally absent in eEF2K−/− and Scn1a ± eEF2K−/− mice. All data are presented as mean ± SEM. N = 4 per group. Statistical analysis ***p < 0.001, ****p < 0.0001 versus corresponding eEF2K−/−; $$$*p* < 0.001, $$$$*p* < 0.0001 versus corresponding Scn1a ± ; %%%p < 0.001 versus corresponding WT; One-way ANOVA, Tukey’s post hoc.** Figure S3. **eEF2K deletion in Scn1a ± mice rescues the level of Akt phosphorylation.** (A)** Western blots analyses and relative quantification of phosphorylated Akt levels in hippocampus (left) and cerebral cortex (right) of 3 months old WT, Scn1a ± eEF2K−/−, Scn1a ± and eEF2K−/− mice. All data are presented as mean ± SEM. WT n = 5 (hippocampus) n = 6 (cerebral cortex), Scn1a ± eEF2K−/− n = 6, Scn1a ± n = 7, eEF2K−/− n = 4. Statistical analysis *p < 0.05 versus corresponding WT, $*p <* 0.05 versus corresponding Scn1a ± ; Kruskal–Wallis test, Dunn’s post hoc for hippocampus and One-way ANOVA, Tukey’s post hoc for cerebral cortex.** Figure S4**. Epileptic Discharges.** (A)** Representative epileptic discharge event (60 s. registration shown) of a Scn1a ± mouse. **(B)** Number of epileptic discharges events calculated in 24 h registration, both in basal condition (WT n = 4, Scn1a ± n = 6 and Scn1a ± eEF2K−/− n = 5) and 7 days after thermal stress (Scn1a ± n = 4 and Scn1a ± eEF2K−/− n = 4). eEF2K deletion protects Scn1a ± mice from epileptic discharges events. Data are presented as mean ± SEM. Statistical analysis for number of epileptic discharges in basal condition *p < 0.05 versus corresponding WT; $p < 0.05 versus corresponding Scn1a ± ; Kruskal–Wallis test, Dunn’s post hoc. Statistical analyses for number of epileptic discharges 7 days after thermal stress condition $p < 0.05 versus corresponding Scn1a ± ; Two-tailed Mann–Whitney test. **(C)** Number of epileptic discharges events calculated in 24 h registration of Scn1a ± mice in basal condition and after 3, 6 and 9 days of treatment with eEF2K inhibitor A484954. Scn1a ± mice present significant reduction of epileptic discharges events at 9 days of treatment when compared with basal condition. Data are presented as mean ± SEM. N = 5. Statistical analysis **p < 0.01 versus corresponding basal EEG; Kruskal–Wallis test, Dunn’s post hoc. Additional file 1:. **Figure S5**. ** (A)** eEF2K−/− mice show no impaired in balance compared with WT mice when cross the 12 mm beam in balance beam test. Data are presented as mean ± SEM. N = 12 per group. Unpaired two-tailed *t*-test. **(B)** eEF2K−/− mice behave like WT mice in pole test measured by the time take to downstream the pole. Data are presented as mean ± SEM. WT n = 7, eEF2K−/− n = 10. Unpaired two-tailed *t*-test. **(C)** eEF2K−/− mice display normal motor coordination when tested by rotarod test. Data are presented as mean ± SEM. N = 10 per group. Unpaired two-tailed *t*-test and two-tailed Mann–Whitney test.** Figure S6. **eEF2K−/− mice show depressed-like behavior but no other autistic behaviors when compared with WT.** (A)** No differences in repetitive grooming behavior evaluated in terms of time spent to do grooming between WT and eEF2K−/− mice. Data are presented as mean ± SEM. WT n = 10, eEF2K−/− n = 12. Two-tailed Mann–Whitney test. **(B)** eEF2K−/− mice show depressed-like behavior in tail suspension test measured by the time spent immobile. Data are presented as mean ± SEM. N = 12 per group. Statistical analysis ***p < 0.001; Unpaired two-tailed *t*-test. **(C-D)** eEF2K−/− mice do not show alteration in recognition memory **(C)** and spatial memory **(D)** evaluated by the discrimination index in the novel object recognition test **(C)** and spatial object recognition test **(D)** at 5, 120 min and 24 h after familiarization phase. Data are presented as mean ± SEM. N = 8 per group in novel object recognition test. WT n = 7, eEF2K−/− n = 5 in spatial object recognition. Unpaired two-tailed *t*-test; Two-tailed Mann–Whitney test (120 min in novel object recognition test). **(E)** eEF2K−/− mice behave as WT mice in social interaction. Social novelty preference index (right). All data are presented as mean ± SEM. N = 5 per group. Two-tailed Mann–Whitney test.** Figure S7**. eEF2k deletion improves visual acuity of Scn1a ± mice.** (A)** Comparison of the time spent to find the food between WT and eEF2K−/− mice (left) and among WT, Scn1a ± and Scn1a ± eEF2K−/− mice (right). Similar behavior between WT and eEF2K−/− mice (left). Data are presented as mean ± SEM. N = 10 per group; Unpaired two-tailed *t*-test. Scn1a ± mice show no differences in spending time to find the food when compared with WT and Scn1a ± eEF2K−/− mice (right). Data are presented as mean ± SEM. N = 10 per group. One-way ANOVA, Tukey’s post hoc. **(B)** Comparison of the ratio of safe choices between WT and eEF2K−/− mice (left) and among WT, Scn1a ± and Scn1a ± eEF2K−/− mice (right). Similar visual acuity for WT and eEF2K−/− mice (left). Data are presented as mean ± SEM. N = 10 per group; Unpaired two-tailed *t*-test. Scn1a ± show a poor visual acuity when compared with WT and Scn1a ± eEF2K−/− mice (right). Data are presented as mean ± SEM. N = 10 per group. Statistical analysis *p < 0.05 versus corresponding WT, $*p <* 0.05 versus corresponding Scn1a ± ; One-way ANOVA, Tukey’s post hoc.** Figure S8**. A484954/pellets mediate reduction of phosphorylated eEF2 in hippocampus, cerebral cortex, liver, kidney and heart of WT mice.** (A)** Quantitative analyses of phosphorylated and total eEF2 show a significantly reduction of phosphorylated eEF2 in hippocampus (left) and cerebral cortex (right) of WT mice after a chronic treatment of 10 days with 1 mg/day dose of A484954 when compared to WT mice treated with placebo. This dose was selected as the only effective in significantly reducing the level of phosphorylated eEF2 compared to the other tested doses (0.5 mg/day or 1 mg/day for 5 days and 0.5 mg/day for 10 days) (data not showed). All data are presented as mean ± SEM. N = 4 per group. No differences in eEF2 expression levels in both hippocampus and cerebral cortex. Statistical analysis ***p < 0.001, **p < 0.01 versus corresponding Scn1a ± treated with placebo; Unpaired two-tailed *t*-test. **(B-D)** Quantitative analyses of phosphorylated and total eEF2 show a significant reduction of phosphorylated eEF2 in liver **(B)**, kidney **(C)** and heart **(D)** of WT mice treated for 10 days with 1 mg/day dose of A484954 when compared with mice treated with placebo. No differences in eEF2 expression levels. All data are presented as mean ± SEM. N = 4 per group. Statistical analysis **p < 0.01, ***p < 0.001 versus corresponding Scn1a ± treated with vehicle; Unpaired two-tailed *t*-test.

## Data Availability

The datasets used and/or analyses during the current study are available from the corresponding author on reasonable request.

## Abbreviations

Na_V_1.1: Type I voltage-gated sodium channel
eEF2: Eukaryotic elongation factor 2
eEF2K: Eukaryotic elongation factor 2 kinase
P-eEF2: Phosphorylate eEF2
WT: Wild type
sIPSCs: Spontaneous inhibitory postsynaptic currents
SMEI: Severe Myoclonic Epilepsy in Infancy
BSA: Bovine Serum Albumin
EEG: Electroencephalography
GABA_A_Rα5: α5 Subunit containing GABA_A_ receptor
Syn2b: Synapsin 2b
