# Supplementary material for: Rescuing epileptic and behavioral alterations in a Dravet syndrome mouse model by inhibiting eukaryotic elongation factor 2 kinase (eEF2K)
Source: Mol Autism. 2022 Jan 3;13:1. doi: 10.1186/s13229-021-00484-0 (PMC8722032; doi:10.1186/s13229-021-00484-0)
Supplement: Supplementary file 1 — Additional file 1: Figure S1. No difference in eEF2 phosphorylation levels in total homogenate of liver, kidney, and heart between Scn1a ± and WT mice. (A-C) Western blots and relative quantification for phosphorylated eEF2 in samples of liver (A), kidney (B) and heart (C) show no significant difference in 3, 6 and 9 months old in Scn1a ± mice compared with WT mice. All Data are presented as mean ± SEM; N = 4 per group. One-sample t-test. Figure S2. Genetic characterization and levels of phosphorylated eEF2 in WT, Scn1a ± , eEF2K−/− and Scn1a ± eEF2K−/− mice. (A) Representative PCR for SCN1A gene (left). WT Scn1a+/+ (WT) mice display single band of 300 bp, heterozygous Scn1a ± mice display one at 300 bp and the other ad 150 bp. Representative PCR for eEF2K gene (right). Length of the bands for WT and KO is at the same high at 1.2 kb. Two different PCR were performed for WT and KO. (B) Representative western blot and relative quantification for phosphorylated eEF2 in hippocampus and cerebral cortex of eEF2K−/−, Scn1a ± , WT (Scn1a+/+ eEF2K+/ +) and Scn1a ± eEF2K−/− show that eEF2 phosphorylation is totally absent in eEF2K−/− and Scn1a ± eEF2K−/− mice. All data are presented as mean ± SEM. N = 4 per group. Statistical analysis ***p < 0.001, ****p < 0.0001 versus corresponding eEF2K−/−; $$$p < 0.001, $$$$p < 0.0001 versus corresponding Scn1a ± ; %%%p < 0.001 versus corresponding WT; One-way ANOVA, Tukey’s post hoc. Figure S3. eEF2K deletion in Scn1a ± mice rescues the level of Akt phosphorylation. (A) Western blots analyses and relative quantification of phosphorylated Akt levels in hippocampus (left) and cerebral cortex (right) of 3 months old WT, Scn1a ± eEF2K−/−, Scn1a ± and eEF2K−/− mice. All data are presented as mean ± SEM. WT n = 5 (hippocampus) n = 6 (cerebral cortex), Scn1a ± eEF2K−/− n = 6, Scn1a ± n = 7, eEF2K−/− n = 4. Statistical analysis *p < 0.05 versus corresponding WT, $p < 0.05 versus corresponding Scn1a ± ; Kruskal–Wallis test, Dunn’s post hoc for hi [file 13229_2021_484_MOESM1_ESM.pdf]

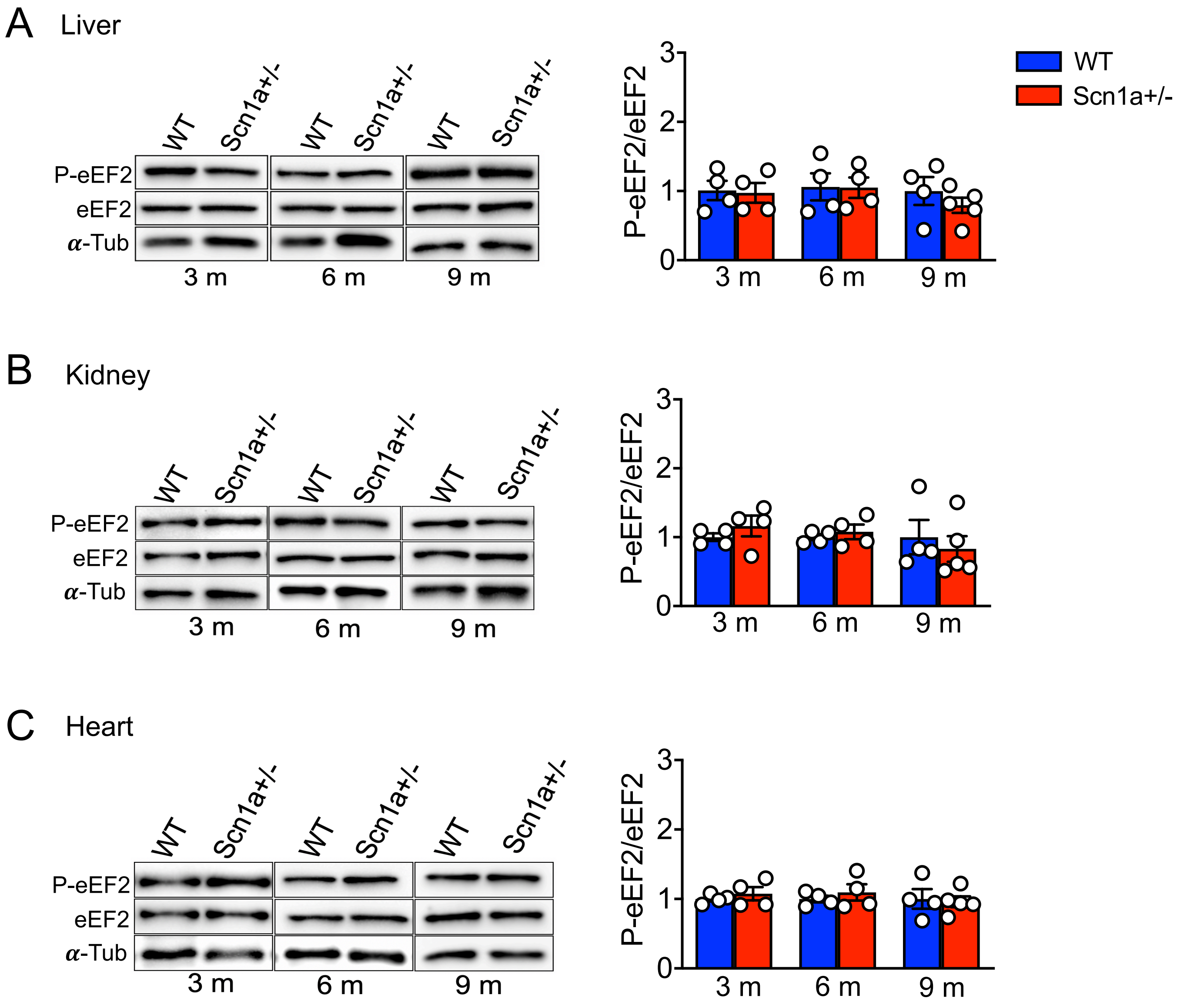

Supplementary Figure 1

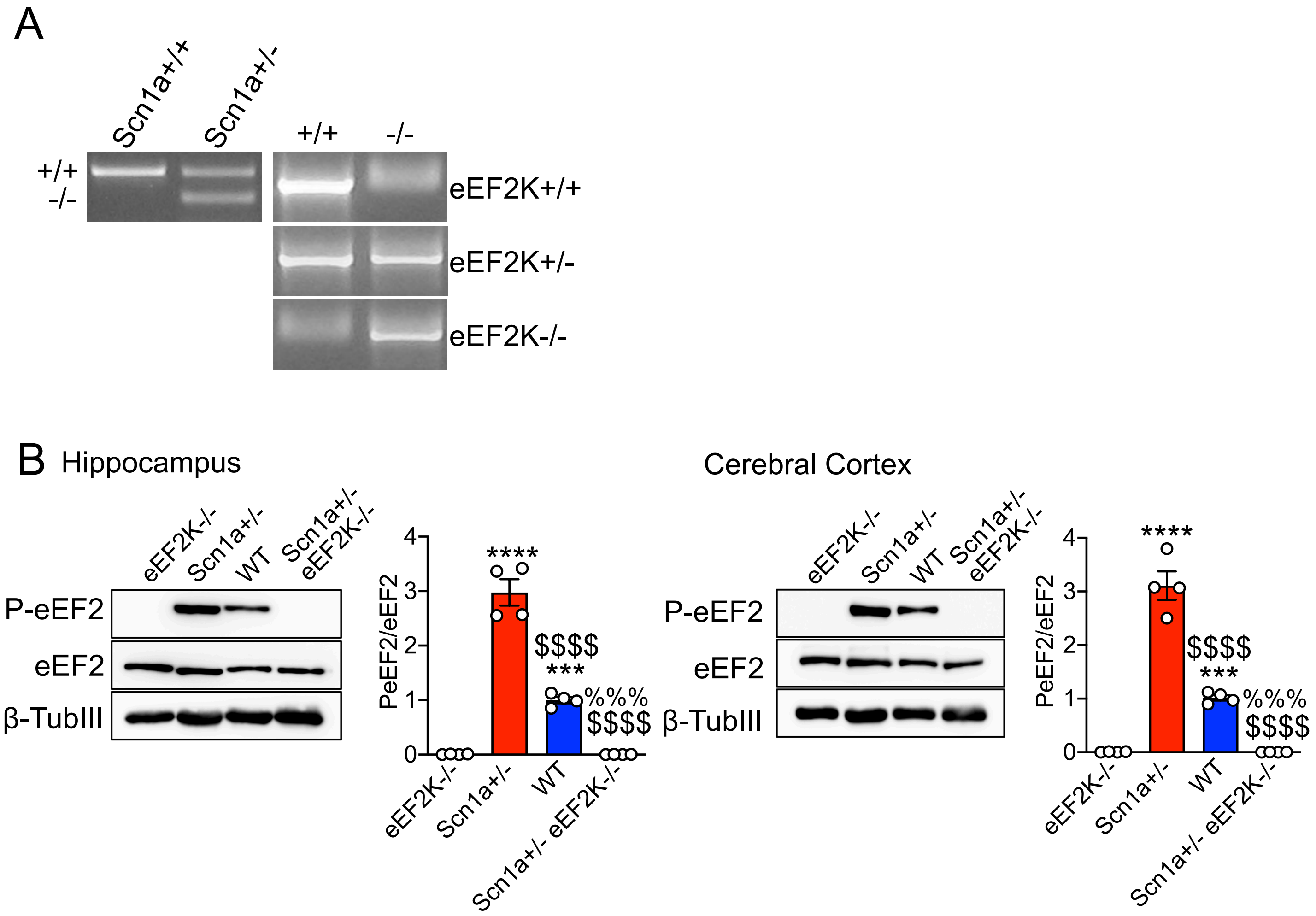

Supplementary Figure 2

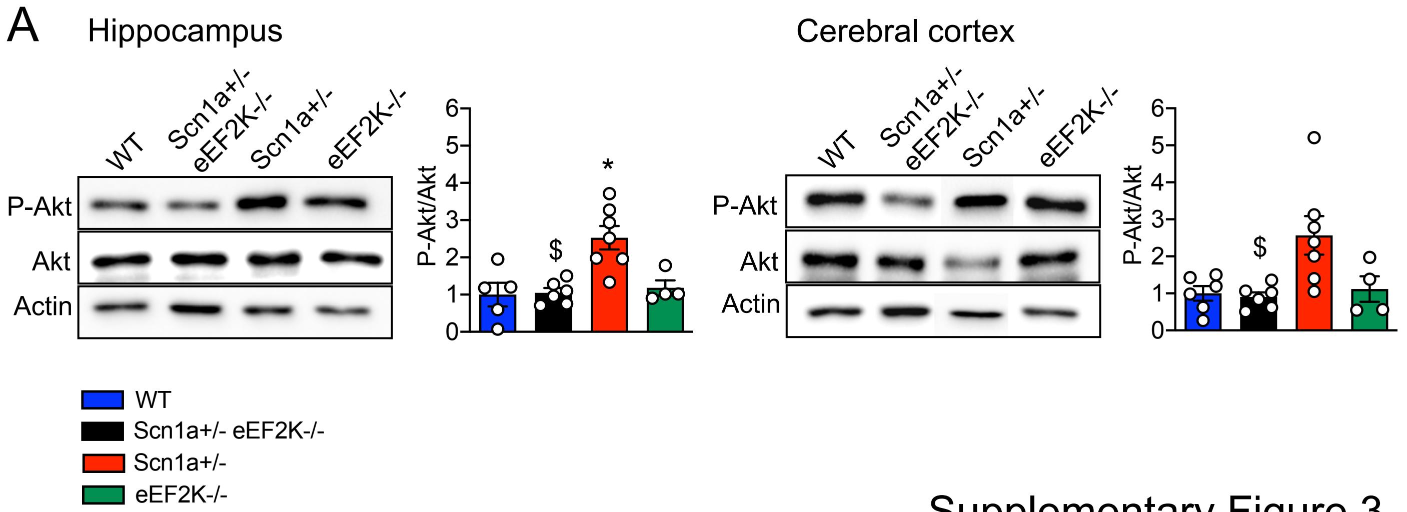

Supplementary Figure 3

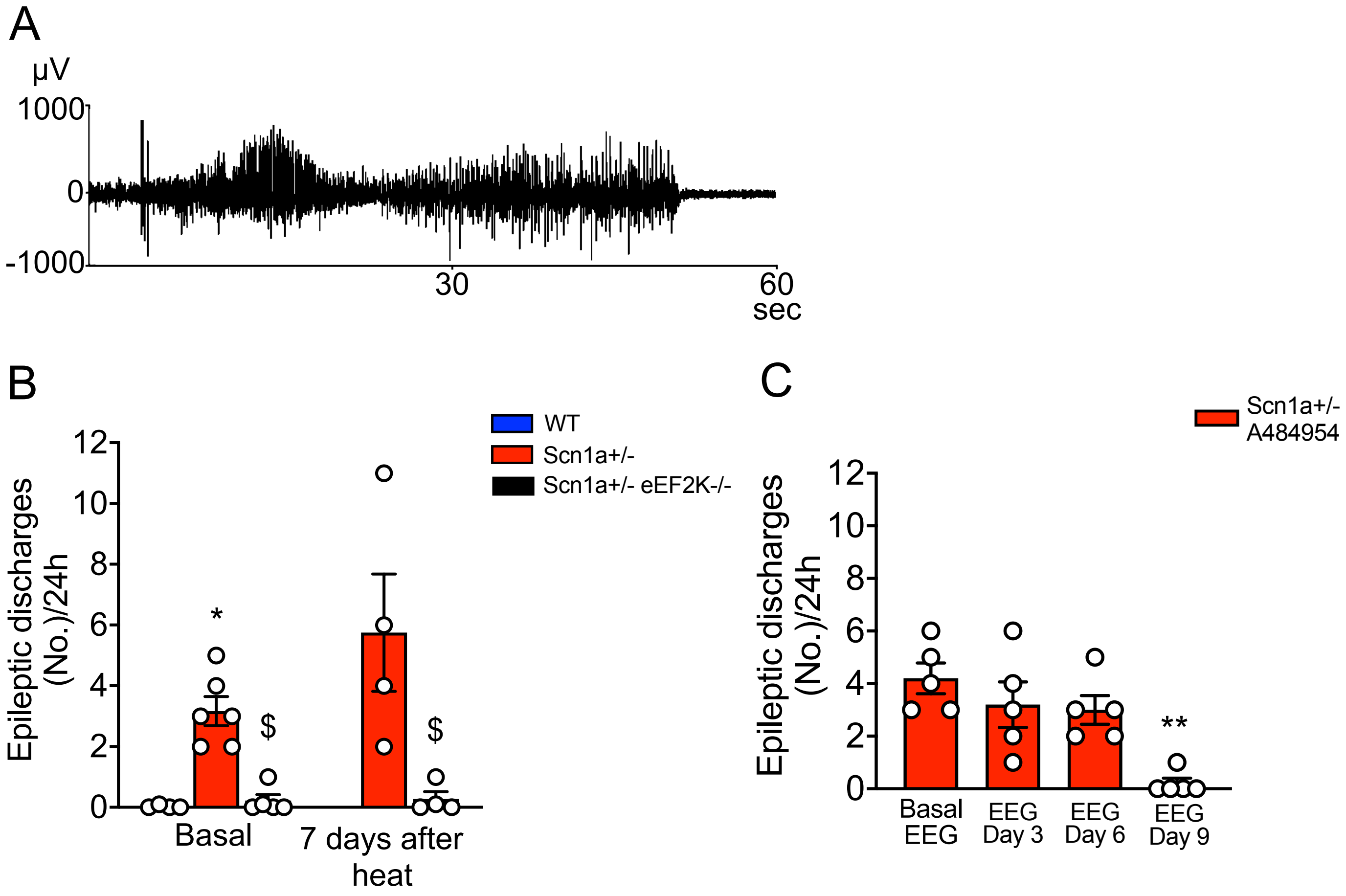

Supplementary Figure 4

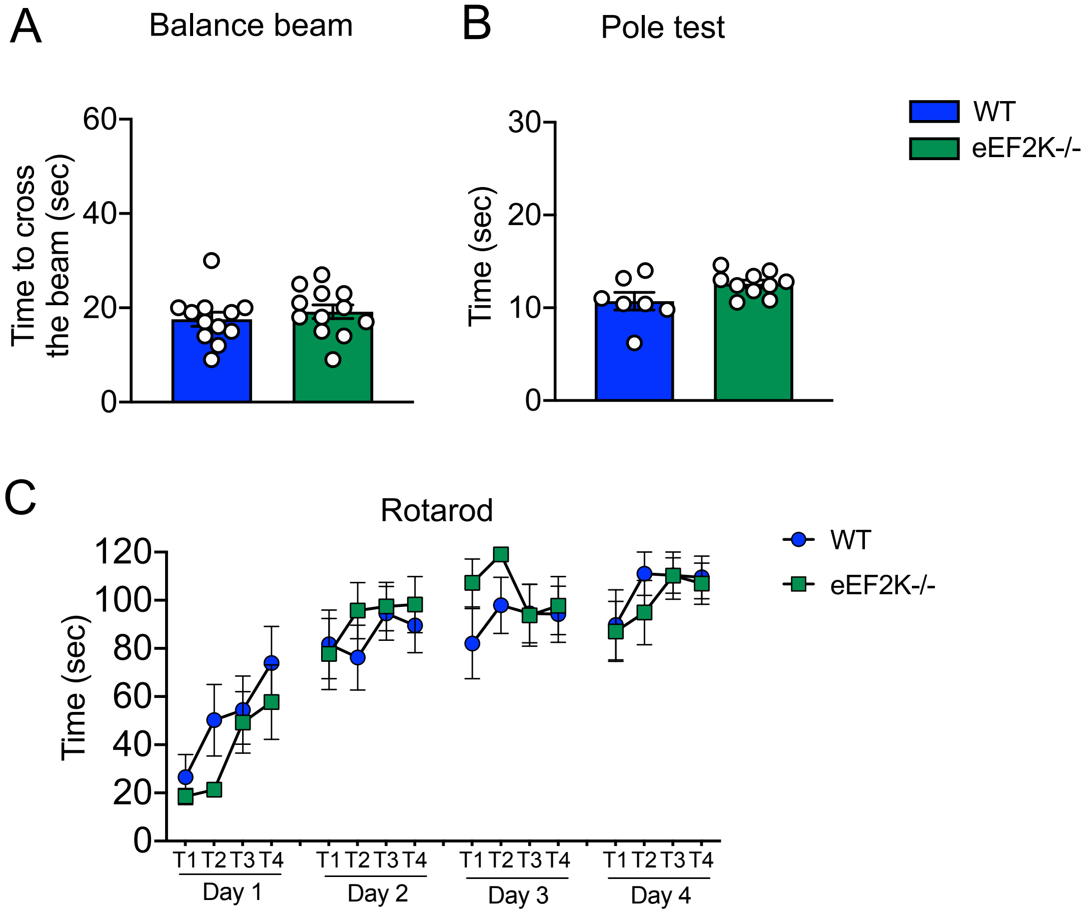

Supplementary Figure 5

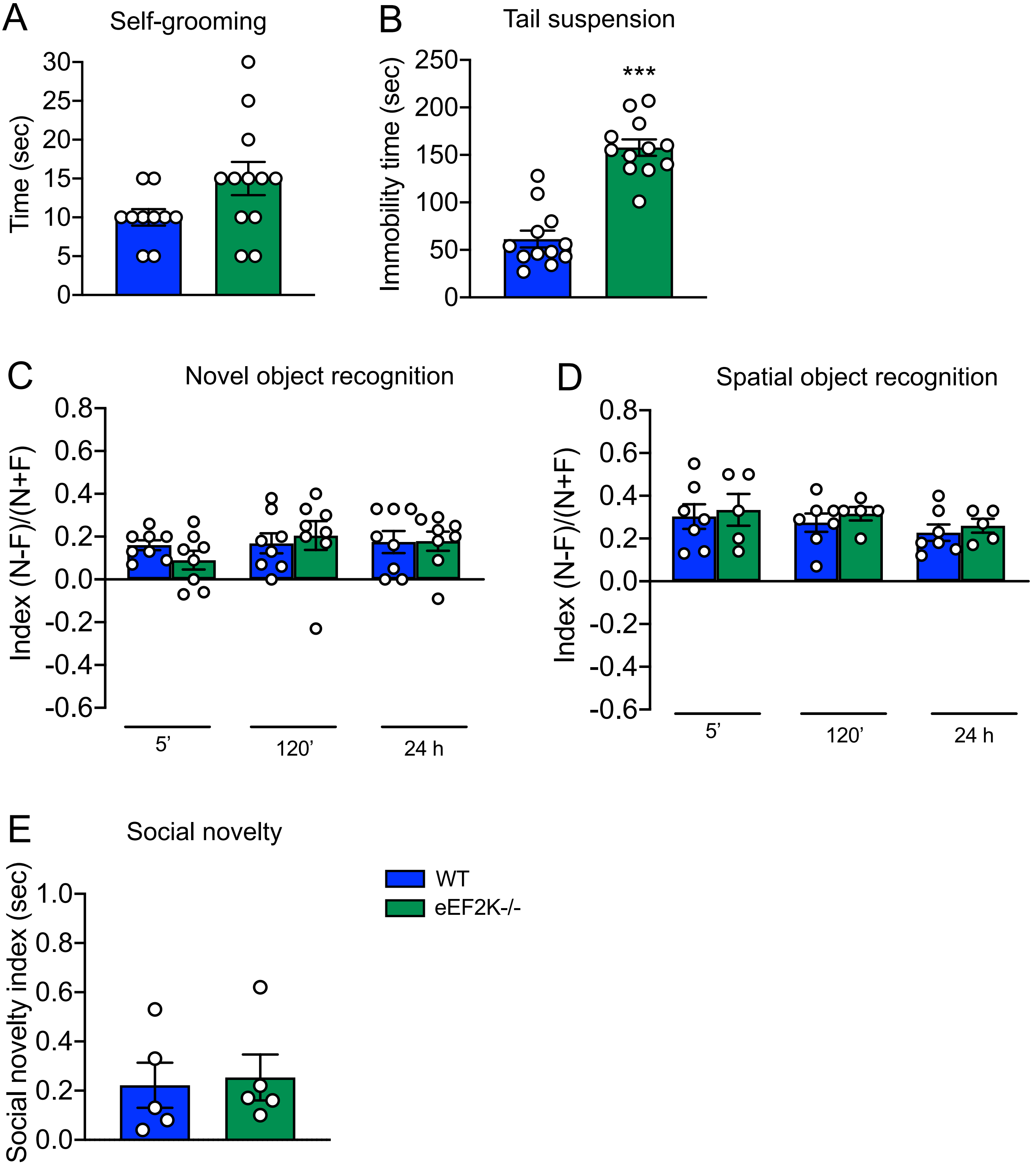

Supplementary Figure 6

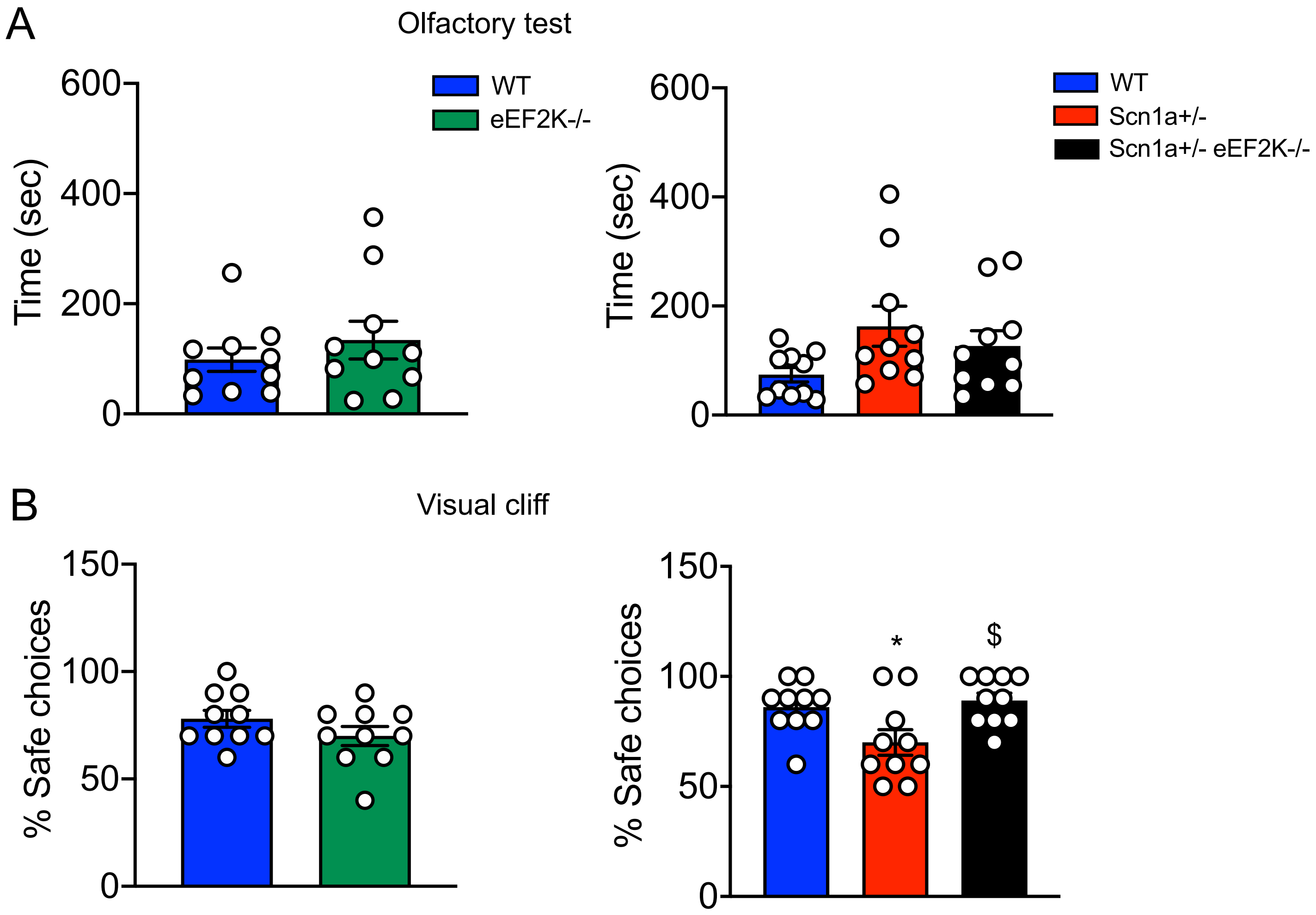

Supplementary Figure 7

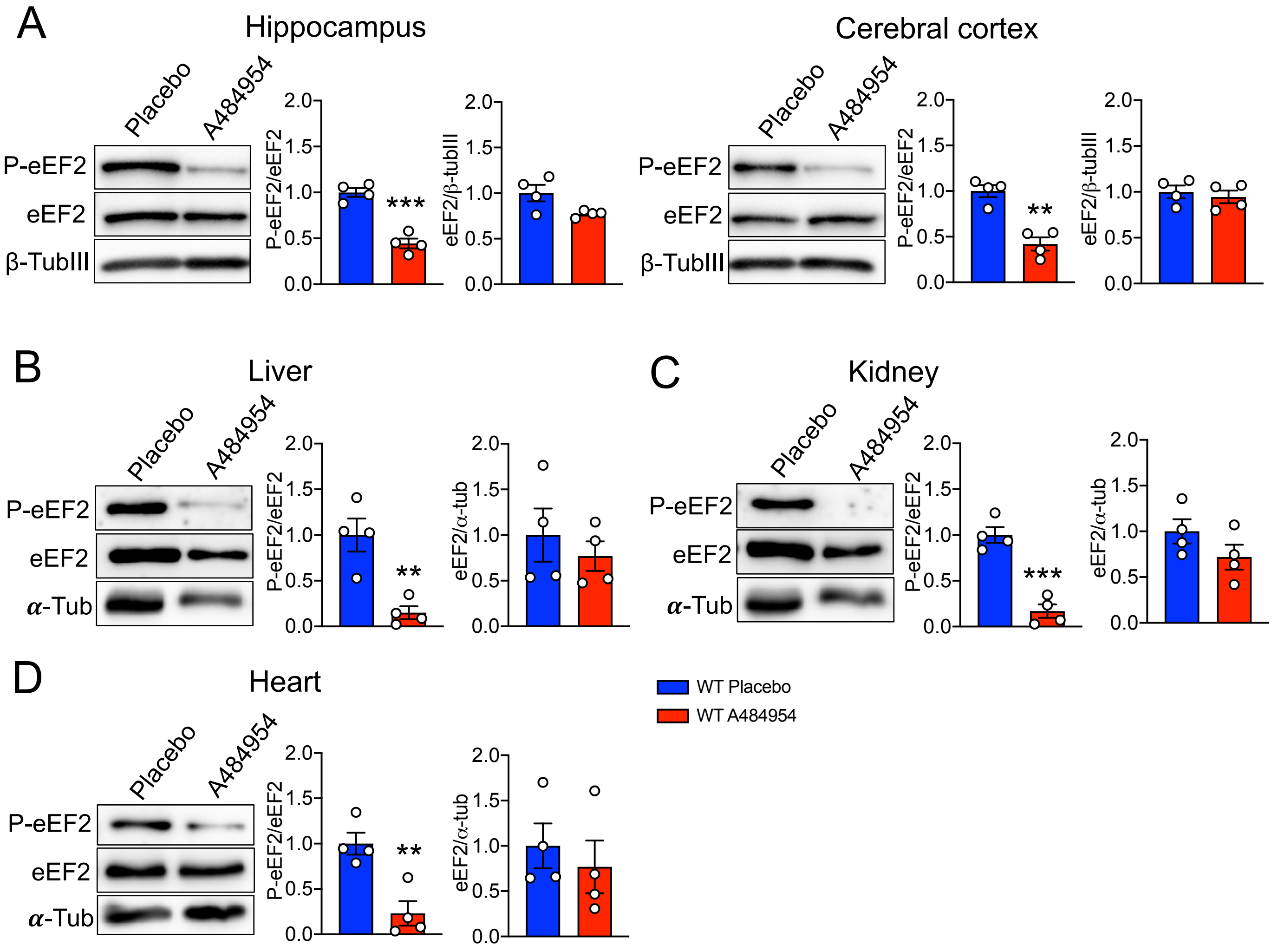

Supplementary Figure 8
